# Aichi Target 18 beyond 2020: mainstreaming Traditional Biodiversity Knowledge in the conservation and sustainable use of marine and coastal ecosystems

**DOI:** 10.7717/peerj.9616

**Published:** 2021-01-04

**Authors:** Paola Fajardo, David Beauchesne, Alberto Carbajal-López, Rémi M. Daigle, L. Denisse Fierro-Arcos, Jesica Goldsmit, Sabine Zajderman, Juan I. Valdez-Hernández, María Yolanda Terán Maigua, Ronaldo A. Christofoletti

**Affiliations:** 1 McGill University, Montréal, QC, Canada; 2 Institut des sciences de la mer, Université du Québec à Rimouski, Rimouski, QC, Canada; 3 Québec Océan, Département de biologie, Université Laval, Québec city, QC, Canada; 4 Natural Sciences and Engineering Research Council of Canada - Canadian Healthy Oceans Network, Memorial University of Newfoundland, St. John’s, NL, Canada; 5 Universidad de Guadalajara, San Patricio-Melaque, Jalisco, México; 6 Charles Darwin Research Station, Charles Darwin Foundation, Puerto Ayora, Galapagos Islands, Ecuador; 7 Institut Maurice Lamontagne, Fisheries and Oceans Canada, Mont-Joli, QC, Canada; 8 Institute of Marine and Environmental Law, University of Cape Town, Cape Town, Western Cape, South Africa; 9 Deep-Ocean Stewardship Initiative, University of Southampton, Southampton, Hampshire, UK; 10 Colegio de Postgraduados, Montecillo, Estado de México, México; 11 Native American Studies Department, University of New Mexico, Albuquerque City, NM, USA; 12 Indigenous Women Network on Biodiversity from Latin America and the Caribbean (RMIB-LAC), Ciudad de Panamá, Panamá; 13 Universidade Federal de São Paulo, Santos, São Paulo, Brazil

**Keywords:** Convention on Biological Diversity, Global Biodiversity Framework, Conservation biology, Indigenous and Community-based Marine Conservation and Protected Areas, Marine and coastal biodiversity conservation, Indigenous Peoples and Local Communities, Aichi Biodiversity Targets, Biodiversity Knowledge Systems, Customary biodiversity conservation and sustainable biocultural use, Traditional Biodiversity Knowledge

## Abstract

Indigenous Peoples and Local Communities (IPLCs) have inhabited coastal areas, the seas, and remote islands for millennia, and developed place-based traditional ancestral knowledge and diversified livelihoods associated with the biocultural use of marine and coastal ecosystems. Through their cultural traditions, customary wise practices, and holistic approaches to observe, monitor, understand, and appreciate the Natural World, IPLCs have been preserving, managing, and sustainably using seascapes and coastal landscapes, which has been essential for biodiversity conservation. The international community has more than ever recognized the central role of IPLCs in the conservation of biodiversity-rich ecosystems, in particular, for the achievement of the Global Biodiversity Targets determined by the Parties to the United Nations Convention on Biological Diversity to tackle biodiversity loss. However, much remains to be done to fully recognize and protect at national levels IPLCs’ Traditional Biodiversity Knowledge (TBK), ways of life, and their internationally recognized rights to inhabit, own, manage and govern traditional lands, territories, and waters, which are increasingly threatened. At the 2018 4th World Conference on Marine Biodiversity held in Montréal, Canada, eight themed working groups critically discussed progress to date and barriers that have prevented the achievement of the Aichi Biodiversity Targets agreed for the period 2011–2020, and priority actions for the Post-2020 Global Biodiversity Framework. Discussions in the “Application of Biodiversity Knowledge” working group focused on Targets 11 and 18 and the equal valuation of diverse Biodiversity Knowledge Systems (BKS). This Perspective Paper summarizes the 10 Priority Actions identified for a holistic biodiversity conservation, gender equality and human rights-based approach that strengthens the role of IPLCs as biodiversity conservation decision-makers and managers at national and international levels. Furthermore, the Perspective proposes a measurable Target 18 post-2020 and discusses actions to advance the recognition of community-based alternative conservation schemes and TBK to ensure the long-lasting conservation, customary biocultural use, and sustainable multi-functional management of nature around the globe.

## Introduction

The richness, abundance, composition and distribution of the Earth’s biological diversity have been substantially declining and changing at unprecedented scales (IPBES, 2019). Unfortunately, the “Biodiversity Crisis”—the accelerated human-induced rates of species loss due to habitat destruction, fragmentation, and degradation—is not widely recognized as a major threat to the Planet and humanity as the threat of climate change despite biodiversity and habitat losses are irreversible in most cases (Hooper et al., 2012; Ceballos et al., 2015; Legagneux et al., 2018). Indeed, the “Biodiversity Crisis” is a major environmental concern from local to global levels due to the adverse effects it is already having on ecosystems (i.e., loss of functional diversity, lower productivity, stability and capacity for the provision of services, such as carbon sequestration) and human well-being (i.e., increased risks to human health linked to zoonoses and reduced food security) (Díaz et al., 2006; Hooper et al., 2012; Ceballos et al., 2015). Urgent and effective conservation actions are needed at a global scale to halt the loss of biodiversity-rich ecosystems, such as marine and coastal ecosystems (Roberts et al., 2002), taking into account multiple knowledge systems (Tengö et al., 2017), and a human rights-based approach. Indigenous Peoples and Local Communities (IPLCs) are the rightsholders of more than 40% of the reminding coastal and terrestrial areas with very low human intervention and biodiversity hotspots, making them central players and decision-makers in biodiversity conservation (IPBES, 2019; Garnett et al., 2018). Yet, IPLCs’ territories, lands, and waters are increasingly threatened. In addition, urgent action is needed to tackle the rapid decline and loss of biocultural diversity (i.e., biological, cultural and linguistic diversity) (terralingua.org), traditional knowledge (TK), and wise practices of IPLCs relevant to the conservation and sustainable use of nature. Biocultural diversity losses have been largely caused by displacements of IPLCs from their traditional ancestral territories, lands, and waters, mainly due to ocean and land grabbing for development projects (Bennett, Govan & Satterfield, 2015; Bavinck et al., 2017), and the establishment of “conventional” private or state-owned Protected Areas (PAs) of restricted use (Mascia & Claus, 2009).

Since its inception 30 years ago, the United Nations Convention on Biological Diversity (CBD) has widely recognized the importance of all types of knowledge systems, including TK systems, to attain its objectives (www.cbd.int/traditional/). At the 2010 Biodiversity Summit, in Nagoya, Japan, Parties to the CBD approved the Strategic Plan for Biodiversity 2011–2020 and its 20 Aichi Biodiversity Targets (www.cbd.int/sp/targets/)—a 10-year framework for global action to tackle biodiversity loss (CBD/COP10/DecisionX/2; www.cbd.int/decision/cop/), which is currently under review to set a new post-2020 framework to be adopted at the 15th Conference of the Parties to the CBD (COP15) (www.cbd.int/meetings/COP-15). Aichi Biodiversity Target 18 is crucially important to IPLCs as it states that ‘*By 2020, the traditional knowledge, innovations and practices of indigenous and local communities relevant for the conservation and sustainable use of biodiversity, and their customary use of biological resources, are respected, subject to national legislation and relevant international obligations, and fully integrated and reflected in the implementation of the Convention with the full and effective participation of IPLCs, at all relevant levels*.’ The conservation of at least 10% of marine and coastal areas through effectively and equitably managed, ecologically representative and well-connected systems of PAs and other effective area-based conservation measures (OECMs) is one of the main goals of Aichi Biodiversity Target 11. These OECMs include Indigenous and Community-based Marine Conservation and Protected Areas (CB-MCAs and CB-MPAs), as referred to in this Perspective.

Given the slow progress towards meeting the Aichi Biodiversity Targets and with the Strategic Plan for Biodiversity 2011–2020 coming to an end, it is pressing to identify both the accomplishments made and the barriers that have prevented the full achievement of the Targets. While recognition of the key role of IPLCs and their TK in biodiversity conservation is expanding internationally, addressing the “Global Biodiversity Crisis” requires respecting the rights of IPLCs—specifically, to own, manage, govern, and inhabit their traditional lands, territories, and waters, and apply their TK and practices. The Post-2020 Global Biodiversity Framework and the 2050 vision “Living in harmony with nature” offer a window of opportunity to frame an effective and ambitious path forward to halt biodiversity loss within the next decade. Therefore, the purpose of this Perspective Paper is to explore Priority Actions to strengthen the recognition of IPLCs as biodiversity conservation managers and decision-makers to inform the current debate around the Post-2020 Global Biodiversity Framework. Priority Actions were selected based on a holistic community-based, gender equality, and human-rights approach for the long-lasting conservation and customary sustainable biocultural use of marine and coastal ecosystems and their biodiversity. This Perspective Paper is structured in four sections: (i) Marine and Coastal Ecosystem Services (ii) TK, Indigenous Peoples and Local Communities, (iii) Convention on Biological Diversity and (iv) Aichi Biodiversity Target 18 beyond 2020: Priority Actions.

## I. Marine and Coastal Ecosystem Services

Oceans cover over 70% of the planet’s surface area and host a variety of marine and coastal ecosystems, such as coral reefs, mangrove swamps, salt marshes and seagrass beds. These ecosystems are among the most biologically diverse and productive systems on Earth, providing multiple essential ecosystem services for human survival and well-being (Roberts et al., 2002; Duke et al., 2007; MA, 2005; Barbier et al., 2011; Ramsar Convention Secretariat, 2013). Some of the critical supporting and regulating services they provide include atmospheric oxygen, climate regulation, nutrient and water cycling, habitat provision, coastal protection and carbon sequestration and storage (Nellemann et al., 2009). Oceans alone produce about half of the world’s oxygen and sequester over 50% of atmospheric carbon dioxide (Nellemann et al., 2009; Liquete et al., 2013). IPLCs living within these diverse ecosystems have maintained close ancestral ties with seascapes and landscapes and marine and coastal biodiversity through their place-based livelihoods, cultural practices, and ways of life (UNEP, 2006; Jokiel et al., 2011). Food, fibres, medicine, firewood and construction materials are vital provisioning services that millions of people, mainly IPLCs, obtain from these ecosystems (Liquete et al., 2013; Fajardo, 2017). Marine and coastal ecosystems also provide multiple cultural services. In the case of IPLCs, cultural services are place-based, namely biocultural heritage, sacred sites, religious and traditional values, sense of place, socio-environmental knowledge, as well as benefits for spiritual, physical, and mental well-being (MA, 2005; UNEP, 2006; Liquete et al., 2013; Pascua et al., 2017). Despite their socio-ecological importance, marine and coastal ecosystems are among the most threatened and exploited areas globally, the latter accounting for 90% of global commercial fisheries (Barbier, 2017). Oceans and coasts are under increasing threat due to large-scale anthropogenic cumulative impacts, such as ocean pollution, acidification and warming, overfishing, illegal wildlife extraction and trade, unsustainable and unregulated large-scale fishing activities, invasive species, habitat loss and coastal development (Jackson et al., 2001; Derraik, 2002; Halpern et al., 2008; Brierley & Kingsford, 2009; Doney et al., 2009; SCBD, 2014b; Vikas & Dwarakish, 2015; Bellard, Cassey & Blackburn, 2016; FAO, 2015a; Laffoley & Baxter, 2016; Halpern et al., 2019). Marine and coastal habitat degradation and loss have been associated with a decline in the quality and number of ecosystem services they provide and in human well-being (Worm et al., 2006; Díaz et al., 2006; Barbier, 2017). These losses and degradation negatively affect IPLCs’ reliance on marine and coastal ecosystems through food insecurity, loss of place-based livelihoods and biocultural diversity, and in certain cases, putting their very survival at risk (UNEP, 2006).

## II. TK, Indigenous Peoples and Local Communities

Indigenous Peoples and Local Communities have developed long-lasting place-based traditional biocultural practices and TK over thousands of years of holistic observations and understandings of their biophysical environment and intrinsic relationships with the Natural World. TK is passed down orally through generations and expanded over the years through adaptive and dynamic social (i.e., institutions), cultural (i.e., oral tradition, customs, practices, stories, art and learning by doing experiences), economic, environmental, philosophical (i.e., worldviews), spiritual (i.e., beliefs) and political processes. For Indigenous Peoples, *Mother Earth* is the fundamental basis of their languages, knowledge, wisdom, and cultural diversity (Teran, 2018). Indigenous People’s biocultural practices and TK are rooted in their close inter-relationships with *Mother Earth* and her ecosystems, and the cosmology that all living things are interconnected and part of nature. TK is described depending on the socio-cultural, ecological and legal context of its application, and there is no internationally agreed-upon definition. Several alternative terms are used to refer to TK, such as Indigenous knowledge (Gadgil, Berkes & Folke, 1993), local and traditional knowledge (Thornton & Maciejewski Scheer, 2012), local ancestral knowledge and practices, traditional ecological/environmental knowledge (Berkes, Colding & Folke, 2000; Usher, 2000; Díaz et al., 2015; Berkes, 2018), traditional wisdom (Turner, Boelscher Ignace & Ignace, 2000), and Indigenous and local knowledge systems (Díaz et al., 2015), among others. In this Perspective Paper, TK refers to the traditional and scientific observational knowledge, practices, innovations, know-how, skills and understandings of IPLCs, which are deeply rooted in their culture, identity, worldviews, relationships, values and beliefs, and pertinent to the conservation and customary biocultural wise use of marine and coastal ecosystems. There is also no official definition of the collective terms “Indigenous Peoples” and “Local Communities” due to the diversity of such communities worldwide. However, it should be noted that the distinction between “Indigenous Peoples” and “Local Communities” is sometimes subjective and challenging depending on the socio-cultural, legal, and political situation among Nations. Indigenous Peoples have distinctive social, cultural, economic, political, organizational and knowledge systems, identities, languages and beliefs, which in many cases have been conserved for millennia (UNPFII, 2019). At the CBD COP12 held in 2014 in Pyeongchang, Korea, the CBD adopted the term “Indigenous Peoples and Local Communities (IPLCs)” in line with the recommendations of the UN Permanent Forum on Indigenous Issues (UNPFII) (CBD/COP/Decision XII/12F) to give more visibility to Indigenous Peoples. Table 1 provides descriptions of the terms “TK”, “Indigenous Peoples” and “Local Communities” as used by international organizations (Martínez-Cobo, 1983; UNESCO, 2017; World Intellectual Property Organization (WIPO), 2018; www.cbd.int/traditional; other descriptions of TK can be consulted in Díaz et al. (2015) and Berkes (2018)).

**Table 1:** Indigenous Peoples, Local Communities and Traditional Knowledge Descriptions.

Organization	IPLCs & TK Descriptions
(UN-DESA) United Nations Department of Economic and Social Affairs: Indigenous Peoples	**Indigenous Communities, Peoples, and Nations:** “Indigenous communities, Peoples and Nations are those which, having a historical continuity with pre-invasion and pre-colonial societies that developed on their territories, consider themselves distinct from other sectors of the societies now prevailing on those territories, or parts of them. They form at present non-dominant sectors of society and are determined to preserve, develop and transmit to future generations their ancestral territories, and their ethnic identity, as the basis of their continued existence as Peoples, in accordance with their own cultural patterns, social institutions, and legal system (p.379)”. “Indigenous Peoples must be recognized according to their own perception and conception of themselves in relation to other groups; there must be no attempt to define them according to the perception of others through the values of foreign societies or of the dominant sections in such societies (p. 368)”. “The right of Indigenous Peoples themselves to define what and who is Indigenous must be recognized (p. 369)”. “No State may take, by legislation, regulations or other means, measures that interfere with the power of Indigenous Nations or groups to determine who are their members (p. 371)” (Martínez-Cobo, 1983)
(ILO) International Labour Organization’s Indigenous and Tribal Peoples Convention (C169)	**Indigenous Peoples:** “Peoples in independent countries who are regarded as Indigenous on account of their descent from the populations which inhabited the country, or a geographical region to which the country belongs, at the time of conquest or colonization or the establishment of present state boundaries and who, irrespective of their legal status, retain some or all of their own social, economic, cultural and political institutions” (Article 1.1). “Self-identification as Indigenous or Tribal is regarded as a fundamental criterion for determining Tribal and indigenous groups” (Article 1.2) (www.ilo.org)
(UNDRIP) United Nations Declaration on the Rights of Indigenous Peoples	**Indigenous Peoples:** “Indigenous Peoples and individuals … have the right to be free from any kind of discrimination, in the exercise of their rights, in particular that based on their Indigenous origin or identity (Art. 1)”. “Indigenous Peoples have the right to self-determination. By virtue of that right they freely determine their political status and freely pursue their economic, social and cultural development” (Art. 3), … “and the right to maintain and strengthen their distinct political, legal, economic, social and cultural institutions, while retaining their right to participate fully, if they so choose, in the political, economic, social and cultural life of the State (Art. 5)” (www.un.org/development/desa/)
(IPBES) The Intergovernmental Science-Policy Platform on Biodiversity and Ecosystem Services	**Indigenous Peoples and Local Communities**: “Ethnic groups who are descended from and identify with the original inhabitants of a given region, in contrast to groups that have settled, occupied or colonized the area more recently.” (ipbes.net/glossary/indigenous-peoples-local-communities) **Indigenous and Local Knowledge Systems (ILK):** “Indigenous and local knowledge systems are in general understood to be dynamic bodies of integrated, holistic, social and ecological knowledge, practices and beliefs pertaining to the relationship of living beings, including people, with one another and with their environments. Indigenous and local knowledge is grounded in territory, is highly diverse and is continuously evolving through the interaction of experiences, innovations and various types of knowledge (written, oral, visual, tacit, gendered, practical and scientific). Such knowledge can provide information, methods, theory and practice for sustainable ecosystem management. Many Indigenous and local knowledge systems are empirically tested, applied, contested and validated through different means in different contexts.” “Maintained and produced in individual and collective ways, Indigenous and local knowledge is at the interface between biological and cultural diversity. Manifestations of Indigenous and local knowledge are evident in many social and ecological systems.” (Annex II to decision IPBES-5/1; ipbes.net)
(CBD) Convention on Biological Diversity	**Local Communities:** Communities that have a long historical association with the lands and waters that they have traditionally live on or used for their subsistence” (UNEP/ CBD/WS CB/LAC/1/INF/5/2016; www.cbd.int) **Traditional Knowledge: “**Refers to the knowledge, innovations and practices of Indigenous and Local Communities around the world”. “Developed from experience gained over the centuries and adapted to the local culture and environment, traditional knowledge is transmitted orally from generation to generation”. “It tends to be collectively owned and takes the form of stories, songs, folklore, proverbs, cultural values, beliefs, rituals, community laws, local language, and agricultural practices, including the development of plant species and animal breeds”. “Sometimes it is referred to as an oral traditional for it is practiced, sung, danced, painted, carved, chanted and performed down through millennia. Traditional knowledge is mainly of a practical nature, particularly in such fields as agriculture, fisheries, health, horticulture, forestry and environmental management in general” (www.cbd.int/traditional/)
(WIPO) World Intellectual Property Organization	**Traditional Knowledge:** Includes the intellectual and intangible cultural heritage, practices and knowledge systems of traditional communities, including Indigenous and Local Communities (traditional knowledge in a general sense or *lato* sensu). TK embraces the content of knowledge itself as well as traditional cultural expressions, including distinctive signs and symbols associated with traditional knowledge. The knowledge resulting from intellectual activity in a traditional context, and includes know-how, practices, skills, and innovations (WIPO, 2018)
(UNESCO) The United Nations Educational, Scientific and Cultural Organization	**Local and Indigenous Knowledge Systems (LINKS):** “Local and Indigenous knowledge refers to the understandings, skills and philosophies developed by societies with long histories of interaction with their natural surroundings. For rural and Indigenous Peoples, local knowledge informs decision-making about fundamental aspects of day-to-day life. This knowledge is integral to a cultural complex that also encompasses language, systems of classification, resource use practices, social interactions, ritual and spirituality. These unique ways of knowing are important facets of the world’s cultural diversity, and provide a foundation for locally-appropriate sustainable development” (UNESCO, 2017)

## III. Convention on Biological Diversity

The CBD is one of the most comprehensive international environmental and biodiversity-related legally binding agreements, whose objectives are the conservation of biological diversity, the sustainable use of its components, and the fair and equitable sharing of benefits arising from the use of genetic resources (www.cbd.int). The Convention works around seven “Thematic Programmes”, which include “Marine and Coastal Biodiversity”, and 27 Cross-Cutting Issues, including “TK, Innovations and Practices (TKIP)—Article 8(j)” and “PAs” (https://www.cbd.int/programmes/), which are relevant in the context of this Perspective Paper. The program of work on “TKIP—Article 8(j)” and related provisions, such as Article 10(c), provides Parties to the CBD with the roadmap to respect, preserve, and maintain TKIP that embodies traditional lifestyles relevant for the conservation and sustainable use of biological diversity. In particular, the Article promotes a wider TKIP application with the approval and involvement of IPLCs and encourages the equitable sharing of benefits arising from TKIP’s utilization (https://www.cbd.int/traditional/). Concretely, Article 10(c) requires Parties to the CBD to protect and encourage customary use of biological resources in accordance with traditional cultural practices. These articles are the rationale behind Aichi Biodiversity 18. Other CBD articles relevant to TKIP include Article 15.5 (i.e., access to genetic resources), Article 16 P(1) (i.e., exchange of information), Article 17.2 (i.e., exchange of results of technical, scientific and socio-economic research, specialized knowledge and TK), and Article 18.4 (i.e., cooperation for the development and use of Indigenous and traditional technologies). Parties to the CBD are required to develop and update National Biodiversity Strategies and Action Plans (NBSAPs) based on the Strategic Plan for Biodiversity and its Aichi Targets. As part of the NBSAPs, Parties communicate their national biodiversity conservation commitments and their pledges to develop sectoral or cross-sectoral national laws, programs and policies, for their implementation and monitoring (Article 6; decision X/2).

## IV. Aichi Biodiversity Target 18 beyond 2020: Priority Actions

**Figure 1:**
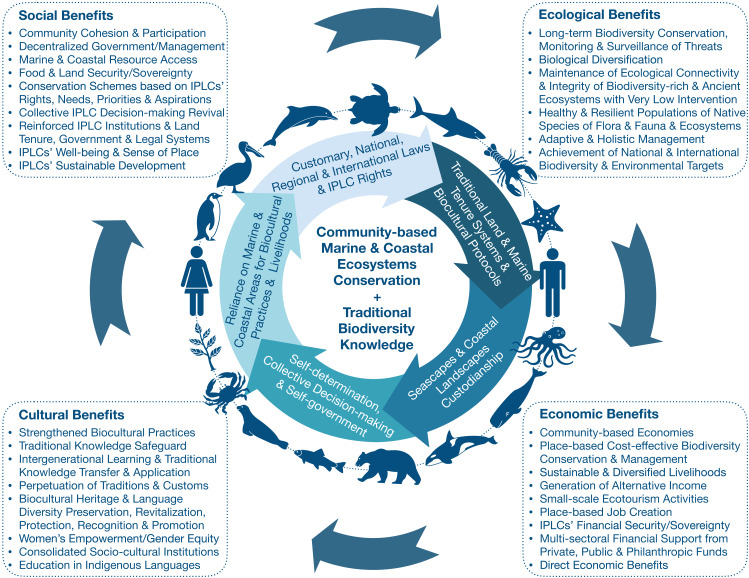
Indigenous and Community-based Marine Conservation and Protected Areas considering harmonic human-nature relationships, as well as gender-balanced social, cultural, economic and ecological benefits.

**Figure 2:**
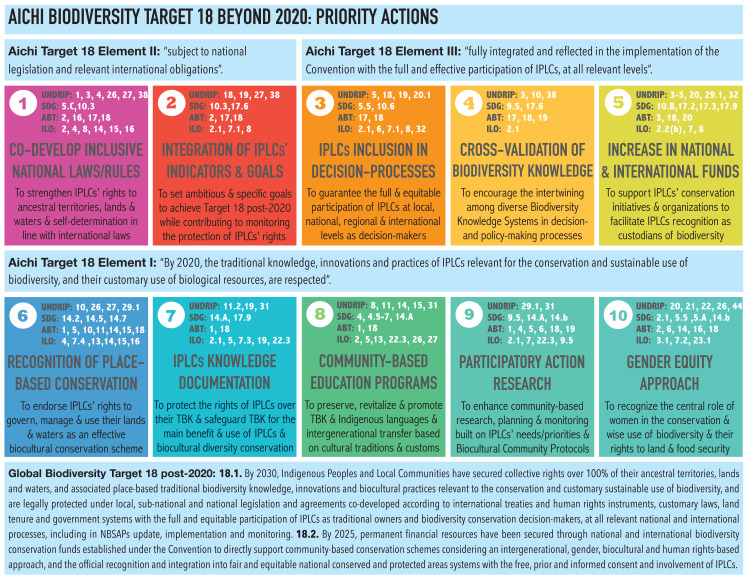
Aichi Biodiversity Target 18 beyond 2020: priority actions. Relevant Articles of International Human Rights Instruments and Targets from International Environmental Agreements are linked to each Priority Action identified: (1) United Nations Declaration on the Rights of Indigenous Peoples (UNDRIP) (A/RES/61/295), (2) Sustainable Development Goals (SDG), (3) CBDs’ Aichi Biodiversity Targets 2010 (ABT) and (4) Indigenous and Tribal Peoples Convention C. 169 of the International Labour Organisation (ILO). Each Article and Target description is included in Fig. S1.

As part of the program of the 4th WCMB held in Montréal, Canada, from 13 to 16 May 2018, participants of eight themed working groups were challenged to reflect critically on the accomplishments of the CBD’s Aichi Biodiversity Targets under the Strategic Biodiversity Plan 2011–2020. Working groups were tasked with identifying 10 priorities for ocean sciences and the conservation of marine and coastal ecosystems beyond 2020. In particular, the “Application of Biodiversity Knowledge” multidisciplinary working group focused its discussions around Aichi Targets 11 and 18, and the equal valuation of diverse knowledge systems referred to in this Perspective Paper as Biodiversity Knowledge Systems (BKS), including Traditional Biodiversity Knowledge Systems (TBKS) of IPLCs. The term Traditional Biodiversity Knowledge (TBK) is used in this Perspective to make a precise distinction with other types of TK, and emphasize its relevance to the CBD. The theme “Application of Biodiversity Knowledge” considered several sub-themes: socio-ecological systems, marine stewardship, education, outreach and participatory programs, and the integration of IPLCs and TK into ocean science, management, and marine policy and law. This Perspective Paper summarizes and discusses the 10 Priority Actions identified as essential to reach Global Biodiversity Target 18 in the post-2020 period and also contributing to Target 11. Priority Actions were selected considering a holistic community-based conservation and sustainable biocultural use approach of marine and coastal ecosystems and their associated biodiversity, along with harmonic human-nature relationships that generate gender-balanced social, cultural, economic and ecological benefits (Fig. 1). Priorities were grouped into the three main elements of the Aichi Target 18 but were not ranked, as specified in Figure 2. For each Priority Action, it is indicated the convergence with relevant articles of international human rights instruments, the Sustainable Development Goals (SDGs), and other Aichi Targets (Fig. 2; Fig. S1). Figure 2 also sets out a suggested measurable Target post-2020 related to IPLCs and their TBK, taking into account the 10 Priority Actions identified.

### Priority #1. Recognizing and incorporating Indigenous Peoples’ rights under international law into local, sub-national, national and regional legislation

The respect, recognition, and integration of TBK and the customary sustainable use and conservation of biodiversity in national legislation and agreements with the full and equitable participation of IPLCs is a critical aspect of meeting Target 18. As a result, the advancement of Targets 11 and 18 post-2020 is strongly dependent on the incorporation of the provisions of international human rights instruments that outline the rights of Indigenous Peoples (Fig. S1), in domestic law and conservation measures. More importantly, these provisions and laws need to be adequately implemented and enforced. This right-based approach should be outlined and considered a compulsory part of the post-2020 Target 18 and related Technical Rational. The UN Declaration on the Rights of Indigenous Peoples (UNDRIP), adopted in 2007, is considered the most complete international resolution on the individual and collective rights of Indigenous Peoples. Other important international human rights instruments that can contribute to advancing Target 18 include the UN International Covenant on Economic, Social and Cultural Rights adopted in 1966, the UN Declaration on the Rights of Persons Belonging to National or Ethnic, Religious and Linguistic Minorities adopted in 1992, and the Indigenous and Tribal Peoples Convention (No. 169) of the International Labour Organization adopted in 1989.

Furthermore, Biocultural Community Protocols developed by IPLCs based on customary, national, and international law are critical legal instruments that could contribute significantly to advancing national and global environmental/biodiversity targets related to IPLCs and their TKIP (naturaljustice.org/community-protocols/). Consequently, Biocultural Community Protocols need to be promoted at the global level to inform governments, and private, research and non-profit sectors of the conditions, responsibilities, and terms for engaging with IPLCs and accessing their knowledge and traditional lands and waters. These Protocols also communicate IPLCs’ values, principles and priorities. While the constitutions and legislation of several countries already recognize the rights of IPLCs to their ancestral territories, there is a serious lack of implementation and enforcement of existing laws and regulations. The lack of enforcement is in part due to limited funding, political will, capacity development, and human resources, as well as significant political changes, generating ownership conflicts over IPLCs’ traditional lands. Well-defined and implemented laws and policies that integrate the provisions of international human rights instruments and Biocultural Community Protocols are needed. Local and sub-national governments have shown willingness to create specific laws and regulations to implement the UNDRIP in their legislation. For example, the Canadian Province of British Columbia in Western Canada introduced Bill 41 to recognize the rights of First Nations to self-determination, self-government, and decision-making. Similar initiatives should be promoted at the global level to develop legal instruments and policies to protect biocultural diversity, TBK, customary conservation schemes, and the rights of IPLCs to have distinct identities and ways of life. For Indigenous Peoples, it is vital to conserve their traditional occupation and ownership rights to ancestral territories, lands and waters, as Indigenous languages, traditional livelihoods and cultural practices are place-based (Martínez-Cobo, 1983). Therefore, for IPLCs, the protection and conservation of *Mother Earth* is a matter of daily life due to their direct reliance on nature and must secure rights over their traditional lands and waters to maintain their close relationships with the Natural World. Correspondingly, nature’s legal rights also need to be considered in national and international legislation, as implemented in Ecuador and Bolivia, as well as in International Conventions, including the CBD and its Global Biodiversity Targets. For example, the 2008 Constitution of Ecuador includes innovative chapters considering new ways of promoting nature-human relationships, a sustainable economy, and well-being: one on the rights of communities, peoples, and nations, and another on the Rights of Nature. In 2011, Bolivia granted legal rights to nature to enhance its environmental protection through the forward-thinking Law of *Mother Earth*. This law sets the principles to establish harmonic and coherent relationships between humans and *Mother Earth* to ensure their well-being, and to guarantee the continuity of the regenerative capacity of all nature’s components and systems.

### Priority #2. Integrating Indigenous and community-based targets and indicators into national and international biodiversity frameworks

As Aichi Target 18 does not set measurable goals, its achievement has not been reached, as measurement and monitoring are challenging. Ambitious, measurable, and inclusive national and international goals to address all aspects of Target 18 are needed in the post-2020 Global Biodiversity Framework. Limited progress to date could also be the result of focusing on only on one or two Target elements and not all, resulting in its partial application. Most Parties have reported limited progress, and current actions are insufficient to achieve Target 18 by 2020 (Leadley et al., 2014; SCBD, 2014a). Although some countries have set specific national commitments for Target 18, such as Costa Rica, Ecuador, and Guatemala, the proposed targets vary significantly among countries depending on their priorities and interests. For example, Costa Rica created a General Mechanism for Consultation of Indigenous Peoples, whereas Ecuador set a target for the development of Biocultural Community Protocols to allow five Indigenous Nations to register their TK associated with biological resources. In comparison, Guatemala set multiple national targets, including the protection of collective TK associated with biological diversity through the implementation of integrated research processes, systematization and legal or *sui generis* protection frameworks. Besides, progress towards Target 18 has been addressed using only four proxies, due to information inaccessibility and variability across countries and communities: (a) IPLCs’ linguistic diversity and number of speakers, (b) land-use change and land tenure, (c) practice of traditional occupations, and (d) the respect, full integration, and safeguards of TK and practices, as well as the full and effective participation of IPLCs in the implementation of the 2011–2020 NBSAPs (SCBD, 2014a).

Determining adequate quantitative and qualitative indicators and criteria to monitor and assess the impact of Target 18 beyond 2020, considering different BKS, expertise, and quality data is of prime importance. Such indicators should include those proposed by IPLCs based on Customary Laws, Biocultural Community Protocols and traditional practices. This Perspective Paper considers that the number of countries redrafting and proposing local, sub-national, national, and regional legal frameworks and policies that recognize the rights of IPLCs could be an effective indicator to measure progress towards Target 18. A key indicator could be the number of agreements co-developed with governments in which IPLCs receive legal ownership of their traditional lands, territories, and waters. Educational, biocultural, environmental health and well-being indicators could also be helpful to assess Target 18. For instance, the number of protected and conserved areas in customary lands run by IPLCs, and sense of place, could be used as indicators. Another indicator could be the number of schools, education centres and universities that implement specialized programs (i.e., environmental education and socio-cultural programs) in Indigenous languages based on the traditions and values of IPLCs intertwined with the respect, conservation, and sustainable use of nature. In many regions, IPLCs do not have access to health services (www.who.int). The lack of health services directly contributes to the reduction in population size of many IPLCs and the number of speakers of Indigenous languages, and the eventual loss of such languages and valuable TBK. It is thus necessary to increase the number of health centres available within communities and provide culturally appropriate medical services in Indigenous languages. Such centres should be designed, approved, and constructed with the support and collaboration of IPLCs and allow the use of traditional medicine. Health indicators could include the number of health centres created, and the reduction of child, women, and elder mortality rates, for example.

Actions at local, regional, and national levels are necessary to draw concrete strategies and measures that ensure the full and equitable participation of IPLCs as biodiversity conservation decision-makers in all NBSAP related processes. IPLCs should be involved in the proposal and selection of National Targets and indicators, and in related monitoring and measurement activities, as well as in the development of inclusive Global Biodiversity Outlooks (GBOs), taking into account the needs priorities, and concerns of IPLCs. Several Indigenous Organizations worldwide are proposing and developing independent strategies to share their own success stories and challenges concerning community-based biodiversity conservation and sustainable use. Some organizations have developed independent indicators to measure the advancement of Target 18 that can be integrated into future GBOs, Biodiversity Frameworks, and NBSAPs. For example, the International Indigenous Forum on Biodiversity created Local Biodiversity Outlooks to document the contributions of IPLCs to the implementation of the Strategic Plan for Biodiversity 2011–2020 (www.cdb.int/gbo), as past GBOs were developed using mainly Western scientific data. Another example is the Indigenous Navigator, a community-based online tool developed for and by Indigenous Peoples to monitor the fulfillment of multiple international human rights instruments, document gaps in their implementation, and monitor the participation of IPLCs in public affairs (nav.indigenousnavigator.com). This online tool was also designed to increase the level of awareness of IPLCs rights and to document the transmission of TK, Indigenous languages, and practices to Indigenous youth (Indigenous Navigator (IN), 2018). Such a platform should be further promoted among United Nations agencies, IPLCs, grassroots organizations, civil society, universities, and governments to have a wider global impact and contribute to monitoring and advancing the CBD’s Global Biodiversity Targets.

### Priority #3. Enhancing and ensuring the full inclusion of Traditional Biodiversity Knowledge and equitable participation of IPLCs in local, national, regional and global biodiversity conservation decision-making and policy-making processes

Through the adoption of Agenda 21 and the CBD at the 1992 Earth Summit, the substantial role of IPLCs in the conservation and sustainable use of biodiversity was recognized with international recommendations for their full involvement in decision- and policy-making processes (UNCED, 1992; www.cbd.int/history/). Despite three decades of recommendations from the international community, efforts to integrate IPLCs and their TBK in decision- and policy-making processes involving national, regional and international biodiversity and environmental assessments and conservation programs have progressed slowly (SCBD, 2014a). Although IPLCs participate in several processes of the CBD, their involvement is considered insufficient compared to the diversity of IPLCs worldwide, due to several underlying factors, including limited government support and recognition, lack of economic resources, language barriers, and gender issues (SCBD, 2014a; Tauli-Corpuz, 2015). There is a need for the translation of information and notifications for IPLCs in their languages, and it is imperative to give IPLC representatives adequate time and space to express their needs and concerns, so that they are effectively represented in decision-making processes during CBD COPs and other such meetings, especially with regard to discussions on the Post-2020 Global Biodiversity Framework. The effective participation and proper representation of IPLCs at the COPs of multiple international conventions could encourage dialogue with local, sub-national, and national governments to further the legal recognition of their rights, including as decision-makers and rightsholders of their traditional lands. A straightforward way to make that possible is identifying and inviting IPLC experts from each signatory country to collaborate in all steps of the Convention processes (Davis & Wagner, 2003), considering participatory and culturally suitable mechanisms. It is also advisable to give IPLCs a central role in the decision- and policy-making process related to the conservation and sustainable management of marine and coastal ecosystems and biodiversity, as well as to food security and sovereignty, water security, traditional fisheries, and other applicable topics.

### Priority #4. Creating mechanisms for Biodiversity Knowledge Systems interface, cross-validation and valuation

There is a growing awareness of the importance of incorporating diverse knowledge systems to address complex environmental challenges and related issues, such as climate change and biodiversity loss, and biodiversity conservation as a whole (Huntington et al., 2002; Anuchiracheeva et al., 2003; Rist & Dahdouh-Guebas, 2006; Mercer et al., 2009; Raymond et al., 2010; Huntington et al., 2011; Thornton & Maciejewski Scheer, 2012; Tengö et al., 2017). Despite this recognition, some barriers still prevent the full consideration and valuation of diverse BKS as equal, which could contribute in part to the slow advancement of the Global Biodiversity Targets. Inadequate coordination among existing knowledge systems, capacity gaps, lack of coordination across governmental agencies and levels of governments, limited participation of IPLCs, and lack of policy-relevant information to support decision-making are some barriers identified in the CBD’s Sustainable Ocean Initiative’s Action Plan (www.cbd.int/soi/). Practical and conceptual differences may exist among knowledge systems (i.e., Western, traditional and citizen science knowledge) (Usher, 2000). Fully integrating and granting diverse BKS the same value in decision-and policy-making processes could be the best approach to identifying appropriate strategies to design and implement effective adaptive biodiversity management and conservation schemes (Cinner & Aswani, 2007), regulations, policies, and long-term research and monitoring programs.

The science-policy interface could be used as a powerful tool to integrate and communicate diverse knowledge systems to inform decision-making at the local, national, and regional levels and incorporate this into global frameworks (Howarth & Painter, 2016). Integrating public participation in scientific analysis can be an important tool for effective science communication, socialization, and decision-making (Dietz, 2013). For example, public prioritizations of ocean benefits such as food provision, coastal protection and biodiversity, have been used to assess ocean health in Canada (Daigle et al., 2017). Improved communication among knowledge holders and users is essential for an effective transfer of diverse knowledge systems at multiple levels using multi-disciplinary methods (Poliakoff & Webb, 2007; Davies, 2008; Bubela et al., 2009; Schäfer, 2009; Fischhoff, 2013). Researchers could adopt new communication and engagement models with stakeholders to encourage or increase openness towards the transfer of knowledge among IPLCs, decision-makers (including IPLCs), scientists, and other stakeholders. The results of scientific research and co-creation of knowledge could be disseminated in ways that are most meaningful to those stakeholders, using communication tools capable of reaching broad and diverse audiences (Groffman et al., 2010; Fischhoff, 2013), such as social media, films and art. As the appreciation of TBK expands, so does the interest in integrating TBK in international processes and agreements. Nonetheless, in previous COPs to the CBD, scientists and IPLCs, have organized separate forums on biodiversity knowledge. Promoting workshops and forums that integrate various BKS could encourage dialogue and trust among the holders of such knowledge for the effective conservation and sustainable use of nature, and provide the basis for future partnerships based on mutual respect and understanding. Guidelines have been proposed to integrate IPLCs into international policy/assessment processes, such as in the UN Intergovernmental Panel on Climate Change (IPCC) assessment reports (Ford et al., 2016). Multilateral organizations, in particular the Intergovernmental Science-Policy Platform on Biodiversity and Ecosystem Services (IPBES), are also taking steps to make this possible. IPBES has proposed a framework to facilitate the input of IPLCs’ comprehensive knowledge on biodiversity and ecosystems into IPBES assessments, giving TK the same value as other knowledge systems and acknowledging IPLCs as key actors in the governance of biodiversity through selection and involvement of IPLCs experts in participatory meetings and dialogues among knowledge systems (Annex II to decision IPBES-5/1; IPBES, 2018). Specific socio-cultural criteria and elements for integrating IPLCs’ traditional, scientific, technical, and technological knowledge have been proposed to identify Ecologically or Biologically Significant Marine Areas (EBSAs) (Convention on Biological Diversity (CBD), 2012). These criteria could be useful to document TBK and community-based conservation and management approaches, and conduct participatory action research with and for IPLCs.

Efforts towards encouraging the integration of TBK in international environmental decision- and policy-making have materialized through specific programs and platforms to document and share TK case studies and best practices (Table 2). One of the oldest initiatives, the Local and Indigenous Knowledge Systems (LINKS) program of the United Nations Educational, Scientific and Cultural Organization (UNESCO), which initially focused on global climate science-policy processes, has joined efforts with the Secretariat of the CBD to advance initiatives to link biological and cultural knowledge. The Secretariat also proposed a TK Information Portal to promote awareness and enhance access by IPLCs and other interested parties to information on TKIP. At COP23, the United Nations Framework Convention on Climate Change (UNFCCC) launched a platform to enhance the engagement of IPLCs in the UNFCCC processes (www4.unfccc). The World Intellectual Property Organization created an Intergovernmental Committee on Intellectual Property and Genetic Resources, TK and Folklore, and an IPLCs Portal to provide access to information for and relating to IPLCs (www.wipo.int/tk). Despite all these initiatives, this Perspective recommends the creation of both national and global platforms to gather and document the available resolutions and recommendations from national and international workshops, forums, and meetings to determine commonalities, avoid duplications and strengthen efforts to effectively integrate IPLCs into international policy and assessment processes.

**Table 2:** International initiatives to document and integrate Traditional Knowledge in policy-processes.

Launch year	Organization	Program/initiative	Objective(s)
2002	UNESCO	(LINKS) Local and Indigenous Knowledge Systems Programme	Promote the inclusion of local and Indigenous knowledge in global climate science and policy processes
N.A.	SCBD	(TKIP) Traditional Knowledge Information Portal	Promote awareness and enhance access by Indigenous Peoples and Local Communities and other interested parties to information on traditional knowledge, innovations, and practices relevant to the conservation and sustainable use of biological diversity. Provide useful and timely information on traditional knowledge and the programme of work for Article 8(j) and related provisions Note: Parties and governments can submit and disseminate through the TKIP, national laws, legislation, policies, programmes, and other relevant information regarding the protection of traditional knowledge. However, the Portal has been inactive since 2015
2010	UNESCO-SCBD Joint Programme	Linking Biological and Cultural Diversity	Advance the implementation of the CBD and deepen global awareness of the interlinkages between cultural and biological diversity
2014	IPBES	(ILK-TF) Task Force on Indigenous and Local Knowledge Systems	Promote effective engagement with Indigenous Peoples and local knowledge holders in all relevant aspects of IPBES work
2017	UNFCCC	(LCIPP) Local Communities and Indigenous Peoples Platform	Strengthen the knowledge, technologies, practices, and efforts of Local Communities and Indigenous Peoples related to addressing and responding to climate change Facilitate the exchange of experiences, best practices, and lessons learned related to climate change mitigation and adaptation in a holistic and integral manner to enhance the engagement of Indigenous Peoples and Local Communities in the UNFCCC process
N.A.	WIPO	Indigenous Peoples and Local Communities Portal	Provide access to information for, and relating specifically to, Indigenous Peoples and Local Communities

### Priority #5. Increasing international funds to support IPLC initiatives and creation of IPLC organizations

The participation of IPLCs in decision-making is critical to promoting the decentralized management of their traditional lands, territories, and waters, and reinforcing their institutions. Granting official recognition to CB-MCAs and CB-MPAs could facilitate direct conservation funding to IPLCs from international organizations, and private, public and philanthropic funds. Being direct recipients could contribute to supporting and safeguarding IPLCs’ community-based conservation, management and government rights, reducing unsustainable practices, limiting the need to sell their lands and to migrate. It could also contribute to avoiding illegal wildlife extraction and trade by creating community-based surveillance brigades and determining effective surveillance methods (e.g., use of cameras, GPS trackers, and local databases to record illegal events). This Perspective suggests the creation of an ‘International Biodiversity Conservation Fund (IBCF)’, similar to the Japan Biodiversity Fund (www.cbd.int/jbf/) and the Green Climate Fund (www.greenclimate.fund/), specifically to support directly community-based conservation efforts and their recognition at national levels. It would be crucial to consider the proposed “IBCF” Fund as part of the Post-2020 Global Biodiversity Framework, taking into account a permanent multi-donor and culturally ad hoc grant funding and capacity development approach. The creation of such an international fund could be an effective strategy to tackle the accelerated loss of biodiversity in many regions by directly granting long-term financing to IPLCs’ organizations without intermediaries in addition to national funds created as part of the NBSAPs. It should be independent of the Voluntary Funding Mechanism created in 2004, at the CBD COP7, to facilitate the participation of IPLCs in the Convention processes, such as in meetings of ad hoc technical expert groups (CBD/COP7/Decision VII/16). Nonetheless, it is imperative to increase the economic input by Parties to the Voluntary Fund to develop clear and effective communication approaches to engage IPLCs as decision- and policy-makers in relevant CBD processes. Creating formal IPLC local, national, and regional organizations could facilitate the receipt of direct funds for their initiatives, such as capacity development, and increase their participation in sub-national, national, regional and international decision- and policy-making processes. Such organizations could represent the common interests, priorities, and needs of communities sharing territories nationally and transboundary, cultural values and principles. The creation of IPLC organizations could contribute to replacing conventional and unsuccessful top-down conservation initiatives, with effective bottom-up conservation initiatives.

Presently, IPLCs are represented in several CBD processes through international organizations made up of members of seven socio-cultural geographical regions determined by Indigenous Peoples under the UNPFII, which are recognized by the Bureau of the COP to the CBD (COP 8 Decision VIII/5). Yet, the inclusion of more IPLC organizations from around the world is needed to guarantee the full and effective participation of IPLCs in CBD processes, taking into consideration a multi-age and gendered perspective, and culturally and linguistically appropriate mechanisms. To illustrate, Indigenous women have organized themselves by establishing their own local and regional organizations and networks to give visibility to their needs and interests at national and international levels (Tauli-Corpuz, 2015). Two organizations that represent women’s interests in the CBD processes are the International Women Network on Biodiversity (iwbn-rmibn.org) and the Indigenous Women Network on Biodiversity from Latin America and the Caribbean. Another important and active organization that has contributed to reinforcing the effective participation of IPLCs in the CBD processes is the International Indigenous Forum on Biodiversity (iifb-fiib.org).

The accreditation of IPLC organizations in biodiversity conservation decision-making processes has reached a historical high. This momentum can further empower IPLCs that lack formal organizations. At the 2016 6th IUCN World Conservation Congress, held in Hawai‘i, the International Union for Conservation of Nature (IUCN) adopted several Resolutions related to Indigenous Peoples. The creation of a new category for IUCN Members, explicitly for Indigenous Peoples’ organizations to include IPLCs in IUCN decision-making processes, was one of the resolutions. This recognition of IPLCs in the international arena could help IPLCs become the direct recipients of multi-sectoral funding sources without the need for intermediaries. Capacity development activities considering the reality and needs of IPLCs (i.e., community organization, writing proposals and administration) are essential to make this possible at a global scale. Such direct funding could further contribute to the creation of research, education, training and cultural facilities within IPLC territories. Facilitating a process for considering IPLCs traditions, cultural practices, values, needs, interests, aspirations and ecotechniques, and management according to their customary laws and protocols. This recognition could also contribute to consolidating and strengthening Indigenous Peoples’ languages, knowledge systems, culture, social, and legal institutions that have been weakened in the past and could also benefit biodiversity conservation (Martínez-Cobo, 1983).

### Priority #6. Strengthening and promoting Indigenous and Community-based Marine Conserved and Protected Areas

Indigenous Peoples and Local Communities all over the world have been conserving, managing and sustainably using marine and coastal areas for millennia and are the traditional rightsholders and custodians of vast well-conserved biodiversity-rich seascapes and coastal landscapes (Ruddle & Hviding, 1992; Veitayaki, 1998; Pollnac, Crawford & Gorospe, 2001; Jokiel et al., 2011; AFN, 2013; Wehi et al., 2013; Garnett et al., 2018). Nevertheless, many barriers continue to prevent official recognition of place-based conservation measures, hinder the protection of IPLC rights, cultures, knowledge systems, customary use of biodiversity. Especially, preventing IPLC involvement in biodiversity conservation and sustainable management, and as decision-makers at local, sub-national, national and regional levels. As a case in point, global environmental concerns, such as biodiversity loss and the protection of marine and coastal ecosystems, have increased pressure on governments to strengthen their commitments to international environmental agreements to meet global biodiversity, conservation, climate change and sustainability targets. Globally, only 7.3% of the world’s oceans and coast are considered “officially protected”, mainly under “conventional” Marine Protected Areas (MPAs) (www.protectedplanet.net), since MPAs are widely accepted policy instruments (Toropova et al., 2010). This in part reflects the fact that governments have largely overlooked the significant potential of officially recognizing already well-preserved seascapes and coastal landscapes through customary and community-based schemes.

More importantly, private or state-owned “conventional” or “formal” PAs—which often see people and nature as separate entities—have on several occasions been unilaterally established within IPLCs’ ancestral and sacred lands, territories and waters. Social, economic, cultural and ecological harm has been caused as a result of expropriation, land acquisition or ocean, coastal, and land grabbing processes while excluding and displacing IPLCs (Coad et al., 2008; Mascia & Claus, 2009; Souza-Andrade & Rhodes, 2012; Stevens, 2014; Worboys, 2015; Ban & Frid, 2018). In addition to mineral extraction, the construction of dams and other major development projects have resulted in changes in land tenure and use, large-scale forced migrations, and displacements, among others. IPLCs are deeply vulnerable to such actions, which affect their integrity, culture, ways of life, identity, and traditional livelihoods, and capacity as custodians of nature (Almudi & Coswig-Kalikoski, 2010; Bennett, Govan & Satterfield, 2015). Strict conservation measures also contribute to the loss of TBK and biocultural practices by prohibiting access to customary fishing areas and natural resources IPLCs rely on for sustaining their traditional ways of life (Coad et al., 2008; Mascia & Claus, 2009; SCBD, 2014a; Bavinck et al., 2017). As a result, traditional livelihoods are converted to illegal activities under MPAs’ regulations and Governmental laws. In general, IPLCs have limited participation in the establishment, government, management, or administration of “official” PAs and rarely get socio-cultural, environmental, economic and political benefits (Bennett & Dearden, 2014; Ban & Frid, 2018). Hence, it is important to make a distinction between who governs PAs (i.e., who decides objectives, holds power, authority and responsibility) and who manages them (i.e., who implements such objectives) due to the socio-cultural, environmental, economic and political implications it has for IPLCs (Borrini-Feyerabend et al., 2013). PAs established within IPLCs territories, lands and waters are frequently governed and managed by government agencies, conservation NGOs, and people external to the area, increasing the costs of conservation and social conflicts. Beyond that, the majority of PAs are financed by governments through limited public funds (Coad et al., 2019), jeopardizing effective biodiversity conservation.

**Figure 3:**
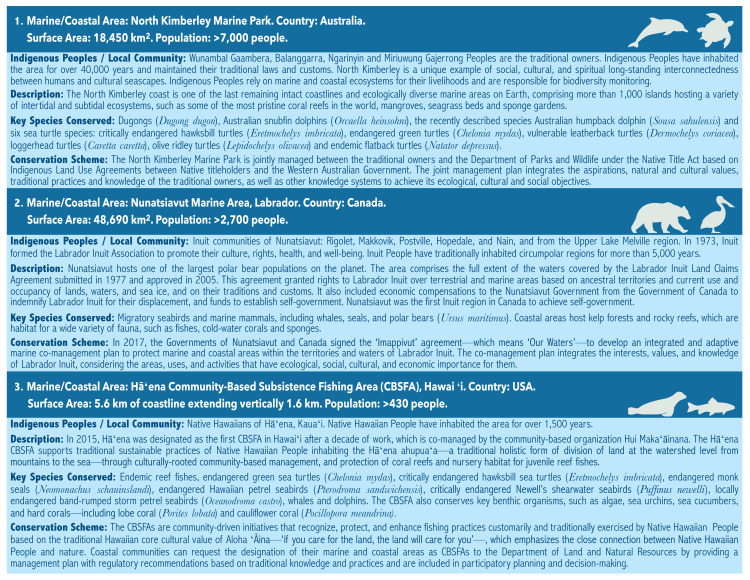
Indigenous and Community-based Marine Conservation and Protection schemes considering the application and valuation of Traditional Biodiversity Knowledge. Sources: (1) Australia: www.dpaw.wa.gov.au; www.wunambalgaambera.org.au (2) Canada: www.imappivut.com; www.rcaanc-cirnac.gc.ca. (3) Hawai‘i, USA: dlnr.hawaii.gov; www.huimakaainanaomakana.org. Biological species global conservation status was obtained from the IUCN Red List of Threatened Species (www.iucnredlist.org), which is the world’s most comprehensive inventory. Only the conservation status endangered or critically endangered is mentioned, including at the local level.

In view of this, community-based conservation offers invaluable advantages for the long-term effective conservation of nature. First, it represents place-based conservation, which reduces administration and operational costs in comparison to “conventional” PAs. Community members can serve as directors, managers, researchers and guards, as many already do under their customary protocols and traditions. The official recognition of community-based conserved areas could allow job creation, strengthen sustainable livelihoods and food systems, and reduce land-use changes. IPLCs have developed a close connection with their lands and waters and hold a unique sense of place, favoring long-term biodiversity conservation and wise decision-making to ensure both nature and community well-being, and socio-ecological resilience. Additionally, this recognition could enable the continuity and permanence of millenary and harmonic relationships between people/communities and nature (Figs. 1 and 3). IPLCs regularly make communal decisions to maintain a balanced use of nature to deliver reciprocal benefits, which have resulted in long-term biodiversity conservation and sustainable biocultural use (Salmón, 2000; Stephenson et al., 2014). Regrettably, in some countries, conservation schemes still consider the displacement and relocation of IPLCs or land purchases of traditional lands instead of strengthening community-based conservation (Almudi & Coswig-Kalikoski, 2010; Tauli-Corpuz, 2015; Samonte et al., 2016). Therefore, the official recognition of CB-MCAs and CB-MPAs could contribute to safeguarding IPLCs lands, territories, waters, and resources from ocean, coastal and land grabbing (IUCN/WCC 2016 Res 088). It could also reduce the pressure on IPLCs to sell or donate their territories to implement conservation, large-scale unsustainable economic developments, or massive tourism projects for public and private interests (Tauli-Corpuz, 2015). Endorsing IPLCs as decision-makers to plan, govern, manage and decree CB-MCAs and CB-MPAs could be a cost-effective strategy to enhance marine and coastal ecosystem conservation, and effective government, management, and monitoring processes in biodiversity-rich areas. Several IPLCs have already been effectively applying this approach as part of their customary laws and had invested resources for biodiversity conservation and monitoring, including human, financial or in-kind resources. Long-term secured Payment for Ecosystem Services and other alternative conservation finance schemes could strengthen IPLCs’ conservation efforts. Local, sub-national and national governments, and private and international initiatives could for instance support and empower IPLCs for the long-term protection, conservation, and management of marine and coastal ecosystems and their biodiversity through the creation of inclusive grant programs. These incentive schemes should involve IPLCs in their design, to guarantee that IPLCs’ rights, needs, and priorities are respected and that taken into account with free, prior, and informed consent.

Furthermore, to fully respect IPLCs’ rights, delimitation of traditional lands, territories and waters, and IPLCs’ reliance on biological resources and interconnectedness with nature, should be assessed, before any attempt to decree new Government or private MPAs, and any development project that could affect IPLCs’ rights, territories, lands and waters. (Simon, 2017). This information can be used to establish or recognize existing CB-MCAs and CB-MPAs. The UNDRIP clearly states that Indigenous Peoples have the right to self-determination, participation in decision-making in matters that would affect their rights, and the lands, territories, and resources which they have traditionally owned, occupied, used, or acquired. It further recognizes Indigenous Peoples’ rights to conserve and protect the environment and the productive capacity of their lands, territories, and resources, and to receive assistance for such conservation and protection, and to determine priorities and strategies for their use or development. A core component of the UNDRIP is the right that Indigenous Peoples have to be consulted and provide free, prior and informed consent of any project affecting their lands, territories, and natural resources, including to the establishment of MPAs. While Aichi Target 11 has progressed substantially, several elements of the Target have not been achieved. Besides, the growing establishment of MPAs corresponds mainly to large-scale ‘conventional’ MPAs of >100,000 km^2^ in remote areas (Leenhardt et al., 2013), and some small-scale MPAs in coastal zones with minimal IPLCs involvement. Hence, more inclusive, equitable, and effective conservation efforts are needed to achieve in full all the elements of Target 11. The CBD and Aichi Target 11 acknowledge that marine and coastal areas should be equitably and inter-culturally managed and recognize OECMs—including CB-MCAs and CB-MPAs. OECMs comprises “a geographically defined area other than a PA, which is governed and managed in ways that achieve positive and sustained long-term outcomes for the in situ conservation of biodiversity with associated ecosystem functions and services and where applicable, cultural, spiritual, socio-economic, and other locally relevant values” (CBD/SBSTTA/22/L.2). The CBD recently adopted several recommendations and guidelines for the consideration of OECMs by Parties to the CBD to advance the achievement of Aichi Target 11 and other international targets (CBD/COP/DEC/14/8).

IPLCs have a critical role in the achievement of future biodiversity and area-based conservation targets. Therefore, it is paramount that their conserved areas traditionally governed and managed receive formal government recognition and support (Corrigan et al., 2018). This recognition would contribute not only to biodiversity protection but to securing IPLCs’ land tenure and sovereignty rights, and increase food security, collective well-being and empowerment of IPLCs (Kothari & Neumann, 2014; Garnett et al., 2018; Ban & Frid, 2018). Recognizing IPLCs as the rightsholders to their traditional lands and waters and respecting their knowledge, innovation, and customary practices must be central to the implementation of Targets 11 and 18 post-2020. The official recognition of OECMs is important, as community-based marine and coastal conservation, management, and government schemes are highly diverse among communities and countries (Worboys, 2015), as are the biocultural and spiritual motivations behind them. In many cases, the protection of sacred sites is the main purpose, rather than biodiversity conservation per se, but such OECMs have contributed to protecting species, habitats, and ecosystems. Area-based conservation measures include: (1) Marine and Coastal PAs, (2) Territories and Areas Governed and Managed by IPLCs, (3) Area-based Fisheries Management Measures, and (4) other Sectoral Area-based Management Approaches (CBD/SBSTTA/22/L.2). Among them, CB-MCAs, CB-MPAs, and Indigenous and Community Conserved Areas (ICCAs) have become widely recognized alternative effective conservation schemes (Pollnac, Crawford & Gorospe, 2001; Johannes, 2002; Worboys, 2015). ICCAs comprise a wide range of IPLCs’ conservation schemes around the world, considered as such based on their cultural value (i.e., territories of life and sacred sites) (iccaconsortium.org). They represent ecologically or culturally significant territories or areas with which IPLCs have established close and deep relationships embedded in their history, social, cultural identity and spirituality. Custodian IPLCs are the principal decision-makers and managers of the proposed ICCAs and should have well-organized and functioning community governance institutions (iccaconsortium.org). ICCAs are proposed directly by IPLCs, including nomadic IPLCs, without having to go through national governments, and voluntarily governed and managed through their customary laws and protocols (ICCA Consortium, 2020). Remarkably, the support to ICCAs has been increasing at the international level due to their potential to contribute to biodiversity conservation and sustainable use, and achieving the Global Biodiversity Targets (Kothari & Neumann, 2014). The German government, the Global Environment Facility, and the United Nations Development Programme (UNDP) set an example by creating a fund to enhance their recognition and support (iccaconsortium.org).

A distinction is made between “conventional” MPAs and OECMs, including ICCAs, as not all OECMs are recognized by governments or included in national systems (Worboys, 2015). Various countries have shown commitment to establishing close collaboration with IPLCs to recognize and decree CB-MCAs and CB-MPAs with shared governance, and subsistence fishing areas guided by their TBK, cultural practices, and values to endorse their customary lands and access rights to marine and coastal areas (Fig. 3). Some countries have also created specific PA categories for community-based MPAs, which are counted as part of their National PAs Systems. For instance, since the 1990s, Australia has been supporting Indigenous Protected Areas (IPAs) to conserve biodiversity and traditional cultural practices, which are managed directly by Indigenous Peoples through voluntary Indigenous Land Use Agreements with the Australian Government in accordance with the Native Title Act, acknowledging IPLCs as landowners and managers, such as North Kimberley Marine Park (DPAW, 2016) (Fig. 3). The IPAs program is co-administered by the National Indigenous Australians Agency. IPAs for account more than 46% of the Australian National Reserve System (www.environment.gov.au). First Nations, Inuit, and Métis in Canada have been governing, conserving, and sustainably using marine and coastal areas over thousands of years through customary laws, and more recently, under multiple conservation schemes, including partnerships with local and federal governments (Stephenson et al., 2014). However, it was until 2017 that the Government of Canada as part of Reconciliation processes committed to working closely with IPLCs based on recognition of rights, respect, cooperation and devolution of traditional lands and waters (Fig. 3). Canada’s Ocean Act and the National Marine Conservation Areas Act officially recognize the relevance of First Nations, Inuit, and Métis and their traditional ecological knowledge to plan, manage and operate MPAs. Other examples include Coastal Marine Spaces for Native People (EMCPO, acronym in Spanish) in Chile (FAO, 2012), and Community-based Management Units for Wildlife Conservation (UMAS, acronym in Spanish) in Mexico, regulated by the Ministry of Environment (SEMARNAT) (Valdez-Hernández, 2016; Fajardo, 2017; Fajardo & Valdez Hernández, 2018). In Hawai‘i, coastal communities have the right to establish Community-based Subsistence Fishing Areas to protect and enhance fishing practices customarily and traditionally exercised for purposes of Native Hawaiian subsistence and culture (DAR, 2014). In Colombia, the Uramba Bahía Málaga National Park is an example of shared governance based on TK (FAO, 2012). Other countries that support community-based MCAs and MPAs include Costa Rica, Ecuador, Fiji, Salomon Islands and Samoa (Veitayaki, 1998; FAO, 2012; Kothari & Neumann, 2014). States could also support OECMs by drafting laws that recognize IPLCs’ traditional lands and waters, by including community-based MPAs and OECMs categories in their national PAs systems, and by creating mechanisms for the devolution of lands and waters to rightsholders. Alternatively, National PAs Institutions could share the economic benefits they receive from tourism and entrance fees with the rightsholders, and local communities living within and near PAs and in buffer zones, contributing to the capacity and sustainable development of IPLCs.

The IUCN recognizes the important role of IPLCs and their cultures in global conservation efforts (IUCN/WCC 2016 Res 075) and supports alternative effective area-based conservation schemes, such as OECMs, including ICCAs overlapped with government-based PAs (Borrini-Feyerabend et al., 2013; IUCN/WCC 2016 Res 030). Specifically, the IUCN has created a PA governance category exclusively for IPLCs with two sub-types: one for conserved areas and territories established and run by Indigenous Peoples and another for Local Communities (Borrini-Feyerabend et al., 2013). However, the IUCN six management and four governance categories may not be sufficient to recognize all types of CB-MCAs, CB-MPAs, ICCAs, and OECMs (Corrigan et al., 2018). An international effort is needed to set a global OECM/ICCA framework with guidelines for their implementation in line with the needs, and priorities of IPLCs, rather than the other way around. ICCAs and OECMs can be directly uploaded by IPLCs to the World Database of Protected Areas (WDPA)—a joint project between the United Nations Environment Programme (UNEP) and the IUCN, managed by UNEP World Conservation Monitoring Centre—to promote their formal recognition (www.unep-wcmc.org). The WDPA can be used to ensure the system protects of IPLCs’ rights to conserve both marine and terrestrial areas within their territories and declare them either as Conserved Areas (www.iccaregistry.org) or PAs (www.protectedplanet.net). The number of hectares returned to their traditional rightsholders could be used as an indicator to measure progress towards Target 18, as applied in Canada with First Nations and Inuit Peoples (Fig. 3). In addition, the number of community-based MCAs, MPAs, ICCAs, and other OECMs recognized or established, already considered to measure progress towards Target 11, could also be an indicator of progress towards Target 18.

Community-based MCAs, MPAs, ICCAs, and other OECMs could also contribute to conserving highly migratory marine species that cross boundaries beyond national jurisdictions by creating collaborative transboundary networks. In the case of PAs, the IUCN identified transboundary governance as a sub-type of the shared governance category (Borrini-Feyerabend et al., 2013). Highly migratory marine species, such as marine turtles, whales, dolphins and sharks, are among the most threatened marine species, and their effective conservation and management require multilateral cooperation and actions. IPLCs could contribute to such efforts through international marine stewardship (Druel & Gjerde, 2014; Lascelles et al., 2014). Indeed, there are several successful experiences of transboundary cooperation among IPLCs from the Pacific Ocean—who share a common history, ancestry, and culture—for the establishment and management of large-scale MPAs through bilateral agreements and collaborative research, monitoring, and enforcement activities (Friedlander et al., 2016). Marine and coastal ecosystems are complex and highly dynamic social-ecological systems that require innovative conservation schemes and ecosystem-based adaptive management (Olsson, Folke & Berkes, 2004; Pikitch et al., 2004; Folke et al., 2005; Armitage et al., 2008; Crowder & Norse, 2008; Katsanevakis et al., 2011; Levin et al., 2012). Adopting a community-based conservation and management approach that promotes IPLCs’ direct governance of marine and coastal areas is key to achieving the goals of the CBD, the Global Biodiversity Targets, and countries’ commitments to multiple international agreements (e.g., the Paris Agreement and SDGs). This community-based approach could also generate numerous socio-cultural, ecological, and economic benefits not only to IPLCs but also to the planet (Figs. 1 and 2).

### Priority #7. Documentation and application of Traditional Biodiversity Knowledge for the benefit of Indigenous Peoples and Local Communities.

Documenting TBK related to the conservation and sustainable use of marine and coastal biodiversity, as well as best practices and lessons learned at local, national, and regional levels is critical, as most efforts have primarily focused on terrestrial ecosystems. The need to integrate TBK in marine and coastal conservation efforts in order to identify key ecological areas for protection and collective environmental solutions was highlighted in various sessions of the 2018 WCMB. IPLCs are likely to have information about spatial/temporal patterns of distribution of species of interest, the location of aggregation/mating areas or nurseries for specific species, stock numbers, identification of critical habitat, and knowledge about the effect of temporal environmental changes on biota (Furgal & Laing, 2012). Hence, TBK can provide valuable information to guide local and national governments’ environmental programs and research projects. At the same time, IPLCs should be included in the decision- and policy-making processes for the application of such TBK for the benefit of IPLCs. There is a need to document the traditional use of marine and coastal species (i.e., food and construction materials), and the interdependence of IPLCs with such species to guarantee their continued customary sustainable use. Similarly, documenting community-based management and conservation systems that have contributed to the preservation of biodiversity is crucial for the establishment and official recognition of community-based MCAs and MPAs, ICCAs and OECMs. It is important to ensure that such knowledge and information is protected, respected, and used appropriately for the main benefits of IPLCs. Thus, there is a need for careful TBK protection, respect, documentation and systematization, and to develop and enforce strict ethical principles and procedures, including for research involving the documentation of TBK by universities, research centres, NGOs, and external individuals/organizations. The creation of community-based and legal frameworks of knowledge ownership to safeguard oral and written TBK at local, national, and international scales is pivotal. Free, prior, and informed consent and involvement of IPLCs is critical to efficiently protect confidential TBK that has been gained and conserved by IPLCs for millennia, and that could be at risk of misappropriation. The “Tkarihwaié:ri Code of Ethical Conduct to Ensure Respect for the Cultural and Intellectual Heritage of Indigenous and Local Communities Relevant for the Conservation and Sustainable Use of Biological Diversity” was adopted at the CBD COP10 in Nagoya to be used as a model by Parties to develop codes of ethical conduct for research, access, use, exchange, and management of information concerning TBK. The Secretariat of the CBD recently published complementary guidelines related to TBK: the Mo’ otz Kuxtal Volyntary Guidelines, the Rutzolijirisaxik Voluntary Guidelines for the Repatriation of TK, and the Voluntary Glossary of Key Terms and Concepts within the context of Article 8(j) and related provisions that can be of use. The Mo’ otz Kuxtal Guidelines provide a framework to ensure fair and equitable benefit-sharing arising from the use of TBK that is, relevant for the conservation and sustainable use of biological diversity, and reporting and preventing unlawful appropriation. It also provides guidelines for the development of mechanisms, legislation, or other related initiatives to ensure a ‘free, prior and informed consent’ or “approval and involvement” for accessing IPLCs’ knowledge, innovations, and practices, depending on national circumstances. In Canada, First Nations have set the OCAP Principles (Ownership, Control, Access and Possession) on how data collection processes in their communities should be conducted, used, protected, and shared (fnigc.ca/ocap), which can serve as a model for other IPLCs. Several universities in Canada have developed protocols and ethics guidelines regarding research involving Indigenous Peoples, such as McGill University (www.mcgill.ca) and York University (research.info.yorku.ca). International organizations, such as the World Intellectual Property Organization, have created guidelines on laws and regulations that should be considered to document and protect TBK (www.wipo.int). Having adequate national legal frameworks according to each country’s circumstances, including *sui generis* systems based upon customary and international law, could prevent the illicit appropriation of TBK by third parties, safeguard its correct use, and ensure adequate benefit-sharing (World Intellectual Property Organization (WIPO), 2004, 2017; UNESCO, 2017).

### Priority #8. Community-based education programs in Indigenous languages

Current official proxies to measure progress towards Target 18 include linguistic diversity and numbers of speakers. Over 370 million Indigenous people live in 90 countries across the world, belonging to 5,000 different groups and speaking more than 6,000 languages (UNPFII, 2019). Nearly half of such languages are disappearing (www.unesco.org) and with them important TBK relevant for biodiversity conservation. The number of speakers of Indigenous languages is also declining. In part due to very small populations of many Indigenous Peoples, which in turn reflects various underlying issues including displacement, lack of services, health problems, and food insecurity, among others (Tauli-Corpuz, 2015). While there is no official figure for the number of people who speak Indigenous languages as a first language, in most communities, it is mainly Elders who speak their ancestral Indigenous languages without passing it on to their children and grandchildren (in Teran (2018)). There is thus a need for initiatives to increase the number of people that can speak both their Indigenous language and the official language of their country, and recognize Indigenous languages as official languages, such as in Mexico. As education programs are often delivered in foreign languages based on Western social and cultural values, there are limited education and work opportunities for young people in ancestral traditions, languages, and traditional ways of life which exacerbates the loss of linguistic diversity and TBK, and attachment to the land (Harrison, 2007; SCBD, 2014a; Simon, 2017).

However, the right of IPLCs to develop and implement education programs that integrate their history, oral traditions, languages, and socio-cultural values is gaining support in many countries mainly through revitalization language programs. In the State of Yucatán, Mexico, Maya people represent 30% of the population, but the number of speakers has been decreasing. The Government of the State of Yucatán through the Institute for Maya Culture Development has launched a program for strengthening and recognizing the Mayan Language and Culture (www.yucatan.gob.mx). In 2019, the Congress approved reforms to the Political Constitution of the State to make obligatory to teach Maya language at the basic educational level. In Cambridge Bay, Nunavut, the Pitquhirnikkut Ilihautiniq/Kitikmeot Heritage Society has been promoting the revival of Inuit culture, language, and history, as well as creating spaces for Elders to exchange their knowledge with youth and children (www.kitikmeotheritage.ca). Another example are the revitalization language programs of First Nations in Canada, which use a language nest model to create language and cultural immersion environments to support fluency in ancestral languages among preschoolers and their parents (www.fpcc.ca/language/Programs/). Alternatively, IPLC initiatives could partner with Colleges and Universities to promote Indigenous language programs for elementary schools, high schools and Indigenous undergraduate students, such as at the Dechinta Centre for Research and Learning in Northern Canada. The Centre has partnered with the University of Alberta, McGill University, and the University of British Columbia to create an innovative program that promotes land learning approaches in Indigenous languages and accredits Elders as professors (Simon, 2017). Environmental science, fisheries, climatology, and marine biology, among other science education programs, could be designed according to IPLCs’ interests, considering gender and age groups in order to build and develop their capacities. The design of specialized local and regional programs should be based on TK, Indigenous methodologies, social, cultural and spiritual values, as well as on their customs and traditions, and implemented in Indigenous languages. Such efforts could contribute to the long-term preservation and revitalization of Indigenous languages and safeguard place-based biocultural diversity. A good example is the Ixil University, in Guatemala, established in 2011 within the “Cuchumatanes” northern highlands—inhabited by the Maya Ixil for more than 2,500 years. The University provides free education based on the concept of ‘Living Well’ (‘TICHAJIL’ or ‘Buen Vivir’), ancestral cultural values, TK, calendars, and history. The University seeks to protect natural environments and Indigenous territories, supports the systematization of scattered knowledge, and develops solutions to some of the problems facing the community, such as land grabbing, mining, and construction of dams.

### Priority #9. Place-based community biodiversity conservation, participatory planning, research, and monitoring

Marine and coastal systems are the most understudied places on Earth (oceanservice.noaa.gov), mainly due to the high costs involved in studying them, limited research funds, complex ecological dynamics, and inaccessibility (St. John et al., 2016). Scientific knowledge gaps are substantial, and high uncertainties exist about long-term ecological processes within marine and coastal ecosystems (UNEP, 2006), and their responses to climate change. Generating knowledge about such complex socio-ecological systems is a continuously adapted learning process that can be supported by multiple knowledge systems (Folke, 2004). IPLCs have developed place-based conservation schemes and methodologies, which apply adaptive management practices and use know-how and learning-by-doing approaches to monitor and sustainably use biodiversity. IPLCs are ideally positioned to make regular direct observations of biodiversity and the environment, as historical and immediate custodians of numerous ecosystems (Berkes, Colding & Folke, 2000; Jokiel et al., 2011; Wehi et al., 2013). IPLCs could be responsible for long-term marine and coastal monitoring and research programs following their customary laws and Biodiversity Community Protocols. IPLCs’ local observations, research methodologies, and TBK could also contribute to generating valuable ad-hoc biological, environmental and socio-ecological information that could help address current environmental challenges in systems with limited data (Harley et al., 2006; Costello et al., 2010; Thornton & Maciejewski Scheer, 2012).

While TBK is well-documented through scientific research programs in some regions, research outcomes may not be necessarily accessible for IPLC decision-making. Therefore, IPLCs should be co-partners in Western scientific research and monitoring programs conducted in their territories. This would ensure research and conservation efforts that consider IPLCs’ needs, concerns and priorities. Participation by IPLCs in collaborative research initiatives could be an effective strategy for enhancing community-based resource management, community organization, and sustainable use practices (Yochum, Starr & Wendt, 2011). IPLCs should receive direct benefits of such research programs, and be co-owners of the knowledge created. Researchers should share, present, and discuss with IPLC authorities research proposals/programs and request free, prior, and informed consent in writing (www.fao.org/indigenous-peoples/). They should also involve community members in their research, including them as co-authors of written materials and sharing research results with the community. The capacities of local leaders and community scientists and technicians should be developed and IPLCs should have the right to create research committees to monitor and participate in research activities. Outcomes of scientific research programs (i.e., peer-reviewed papers, books, reports) conducted in IPLC territories and about their communities should be accessible to IPLC authorities (CBD Article 17.2) as such information could be beneficial for IPLCs in developing community-based management plans, and in decision- and policy-making processes. There is a growing interest among IPLCs, including their youth, in participating in community-based research and monitoring of natural resources. Research programs can be developed and supported following Indigenous and local “research” and monitoring protocols to enhance self-determination and autonomy, which could be used as an indicator to advance Target 18 post-2020. IPLCs’ leadership and participation in research programs can be promoted by applying a Participatory Action Research approach, participatory mapping (e.g., SeaSketch), and integral planning tools. Participatory approaches can lead to collaborative partnerships among multiple stakeholders for the conservation of biodiversity, and the development of integral assessments to address socio-ecological issues affecting IPLCs’ lives (Rockloff & Stewart, 2004; Merrifield et al., 2013; Teixeira et al., 2013; Pita et al., 2016; Teran, 2018).

Citizen science, an innovative approach with benefits for environmental and social sciences, could also contribute to linking traditional and scientific knowledge and help co-produce useful data for the development of integrated and intercultural management conservation policies. Citizen science programs could for example, strengthen the participation of IPLC youth in scientific data collection via sampling, sorting, and species identification (Darwall & Dulvy, 1996; Foster-Smith & Evans, 2003; Delaney et al., 2008; Bonney et al., 2009; Goffredo et al., 2010; Bonney et al., 2014). While projects involving citizen science for biodiversity monitoring focus mainly on terrestrial ecosystems, there have also been some marine and coastal projects (Branchini et al., 2015; Stuart-Smith et al., 2017; Vincent et al., 2017), as well as multilateral efforts such as the Ocean Sampling Day (www.assembleplus.eu). The creation of local, sub-national, national and regional biological databases could also further support the conservation of marine and coastal areas and their biodiversity. Such databases should consider both scientific and TBK following Biocultural Protocols developed by IPLCs, such as the OCAP developed by First Nations from Canada.

### Priority #10. Including gender participatory approaches to assess the role of women in marine and coastal biodiversity, conservation, sustainable biocultural use, and food security and sovereignty

Women have traditionally had an essential role in caring for their families, community well-being, and maintaining the sustainability of community food systems, and in the conservation and sustainable use of nature in general (FAO, IFAD, UNICEF, WFP & WHO, 2019). Women are traditional seed keepers and growers (Winniefridah & Manuku, 2013), developers of cultures, and protectors of languages and biodiversity knowledge (Shiva, 1992); however, these roles are often overlooked in research, conservation, and sustainable development programs (Stacey et al., 2019). Coastal communities are highly dependent on marine and coastal environments and have developed site-specific diversified livelihoods, including traditional artisanal fishing. Successful Indigenous and community-based traditional artisanal fishing approaches provide practical examples of how TBK could be applied in marine and coastal conservation, management and sustainable use (Wilson, Raakjær & Degnbol, 2006; Eddy, Gardner & Pérez-Matus, 2010; Leite & Gasalla, 2013; Stephenson et al., 2014; Brewer & Moon, 2015; FAO, 2015b: Fischer et al., 2015). However, most studies on traditional marine resource management focus on fisheries and the role of men. It is critical to determine the biodiversity conservation role of women and their dependency on marine and coastal resources for their livelihoods, including traditional fishing activities, to assess their needs (Salafsky & Wollenberg, 2000). Many women are heads of households and practice traditional fishing for their families’ subsistence, however this role is not widely recognized and documented (Fitriana & Stacey, 2012; Matsue, Daw & Garrett, 2014). Understanding the role and contribution of women to the protection and wise use of marine and coastal biodiversity could provide socio-ecological and cultural fundamental information required to empowering women, improving their livelihoods, and increasing their representation in decision-making processes (Alami & Raharjo, 2017). Involving women in biodiversity conservation and management could substantially contribute to eliminating hunger and malnutrition while preserving cultural practices and increasing community support, as Indigenous women and girls are likely to experience more food and nutrition insecurity than men (Aswani & Weiant, 2004; Fitriana & Stacey, 2012; Matsue, Daw & Garrett, 2014; FAO, IFAD, UNICEF, WFP & WHO, 2019).

The United Nations 2030 Global Agenda for Sustainable Development recognizes the need to address global socio-environmental challenges and implement interdisciplinary approaches and strategies. The SDGs are designed to encourage cross-sectoral, multi-stakeholder collaboration to improve human well-being (i.e., reducing social and gender inequality), preserve nature, and tackle climate change, and are highly linked to the Aichi Targets (www.cbd.int/development). The SDG approach can be used as a model for setting the new Global Biodiversity Targets and indicators which should be closely linked with the SDGs. Achieving gender equality to access land and productive resources for women and girls to halt hunger and malnutrition is the direct focus of several SDGs and related targets, such as SDG #1.4 (i.e., equal rights to economic resources, ownership, and control over land), SDG #2.2 (i.e., end all forms of malnutrition), SDG #2.3 (i.e., increase the income of small-scale food producers), SDG #5 (i.e., promote gender equality and empower all women and girls) and SDG #5.5 (i.e., ensure women’s participation and leadership in decision-making) (sustainabledevelopment.un.org). The conservation and sustainable use of oceans, seas, and marine resources is the objective of SDG #14. In 2018, the UN proclaimed a Decade of Ocean Science for Sustainable Development beginning in 2021 based on SDG #14 and the first World Ocean Assessment to support global efforts to reverse the cycle of decline in ocean health (oceandecade.org). The SDG #14 is linked to CBD efforts to conserve and sustainably use oceans and coasts, such as the Sustainable Ocean Initiative (www.cbd.int/soi/). Mainstreaming TBK and community-based conservation taking into account both livelihoods and women-centred approaches that recognize the equitable distribution of roles of Indigenous men and women in the conservation and customary sustainable use of marine and coastal ecosystems is fundamental. Such an approach could contribute to addressing several socio-economic, cultural and ecological issues, such as biodiversity conservation, access to natural resources for livelihoods and food security, well-being, land tenure, and sovereignty at local, national, and regional levels (Nursey-Bary, 2010).

## Key messages

- This Perspective highlights the need to further multidisciplinary and culturally appropriate efforts across different governance levels to recognize and engage IPLCs as key biodiversity conservation and land-use decision-makers in local, sub-national, national, regional and international decision- and policy-making processes that affect their rights, TBK, ancestral territories, lands and waters, and well-being.
- Mandatory inclusive cross-sectoral actions, legislation, policies, and agreements co-developed with the full and equitable participation of IPLCs based on international human rights instruments, customary laws, and traditional land tenure and government systems are needed to recognize, respect, and protect IPLCs’ rights to govern, manage and use their ancestral territories, lands, and waters, and to self-determination. At the same time, it is urgent to support, strengthen, and promote successful place-based community conservation schemes and traditional sustainable biocultural practices, and protect IPLCs’ TBKS, languages, and ways of life.
- Halting the biodiversity crisis requires profound societal, legal, and institutional transformative changes that boost both individual and collective awareness of the importance of nature to humanity, which is indispensable for the sustainable use and protection of healthy and resilient ecosystems and their biodiversity. The ten priorities identified in this Perspective could serve as a roadmap for governments, organizations, decision- and policymakers, and other relevant actors to embrace an inclusive and holistic rights-based community conservation approach as a cost-effective strategy to ensure the long-lasting preservation of the remaining biodiversity-rich and ancient ecosystems with very low human intervention.
- Ongoing negotiations of the Zero Draft of the Post-2020 Global Biodiversity Framework and the upcoming UN Biodiversity Conference (CBD COP15) offer an opportunity to Parties to the CBD to reinforce their commitment and leadership to craft an effective global path forward to prevent further habitat and biodiversity losses and safeguard Key Biodiversity Areas. The pathways towards biodiversity conservation must reflect ambitious post-2020 Global Biodiversity Targets and national agendas aligned with the 2050 vision of “Living in harmony with nature”, which will likely determine the fate of the diversity of life on Earth.
- While several countries have advanced the recognition of IPLCs’ rights, political will or significant political changes will likely influence how these perspectives and future biodiversity targets can be achieved. Therefore, keeping a Global Biodiversity Target exclusively for IPLCs, TBK, community conservation schemes, and the customary use of biodiversity in the Post-2020 Global Biodiversity Framework with measurable goals and culturally appropriate implementation and monitoring mechanisms is paramount.
- Involving IPLCs as biodiversity conservation decision-makers in the negotiations of the post-2020 framework is critical for setting more ambitious, inclusive, measurable, and well-defined Global Biodiversity Targets and participatory implementation mechanisms that take into account intergenerational, gender equity and rights-based approaches. Besides, it is crucial to include IPLCs as central actors and decision-makers in the update, implementation, monitoring, evaluation, and review of local, sub-national, and national biodiversity targets and action plans, and in the design of conservation policies and programs.
- Although some quantitative indicators have been adopted to measure progress towards achieving Target 18, they refer mainly to linguistic and land ownership issues. A holistic scope is necessary to identify and include quantitative and qualitative education, culture, health, and well-being indicators that consider diverse BKS.
- The official recognition, support, and promotion of community-based conserved areas and other IPLC biodiversity conservation schemes and their inclusion into fair and equitable national conservation and PAs systems could contribute to not only reaching national and global biodiversity conservation goals but also climate change, sustainability, and social equity targets, and to preserving diverse TBKS and biocultural diversity. Therefore, it is essential to establish an International Biodiversity Conservation Fund along regional and national multi-donor funds to exclusively and directly support place-based community conservation without intermediaries, as recommended in this Perspective.
- Facilitating, enhancing, and promoting equitable cross-validation and valuation mechanisms for diverse BKS, including TBKS, and knowledge co-creation processes are necessary to tackling biodiversity loss and climate change from local to global levels. The valuation of diverse BKS could benefit the design of inclusive and culturally ad hoc conservation and financial schemes, environmental policies, research, education and gender equity programs.
- Additionally, it is necessary to increase the funds of the Voluntary Funding Mechanism to ensure the full and equitable participation of IPLCs in the development of Global Biodiversity Frameworks and Outlooks and in all relevant processes of the Convention. Including to cover the fees of interpreters and translators for IPLC full and effective participation in related decision-and policy-making and communication processes.
- Given the increased number of efforts and programs of several international organizations and intergovernmental bodies, including the CBD, FAO, IPBES, UNESCO, and UNFCCC, to engage IPLCs and integrate TK in their respective processes and assessments, and biodiversity conservation, this Perspective recommends bolstering multilateral collaborative initiatives at local, sub-national, national, and regional levels through capacity development and promotion of multidisciplinary and cross-sectoral dialogues.
- Furthermore, this Perspective emphasizes the importance of creating and interlinking national, regional, and global platforms specifically designed to gather documents, make accessible, and communicate the resolutions and recommendations resulting from national and international workshops, forums, webinars, and meetings on issues related to IPLCs and TBK.
- Such interlinked platforms are indispensable to determine commonalities, reduce time-efforts, and avoid work duplications and could have a significant influence if translated into multiple languages, including Indigenous languages. These collaborative initiatives could further help communicate and advance international environmental agreements and assessments by fostering multilateral cooperation while guiding Parties, organizations, scientists, and other stakeholders on how to mainstreaming TBK, biodiversity conservation, and IPLCs’ rights across different sectors and government systems.

## Supplemental Information

10.7717/peerj.9616/supp-2 Supplemental Information 1 Description of Articles and Targets included in Figure 2.

## Additional Information and Declarations

### Data Availability

The following information was supplied regarding data availability: This study did not generate any raw data as it is a Perspective Paper.
