# Supplementary material for: Aichi Target 18 beyond 2020: mainstreaming Traditional Biodiversity Knowledge in the conservation and sustainable use of marine and coastal ecosystems
Source: PeerJ. 2021 Jan 4;9:e9616. doi: 10.7717/peerj.9616 (PMC7852408; doi:10.7717/peerj.9616)
Supplement: Supplemental Information 1 [file peerj-09-9616-s001.pdf]

Summary of Relevant Articles of International Law Instruments and Targets from International Agreements linked to each Priority Action identified, as shown in Figure 2.

Articles of the United Nations Declaration on the Rights of Indigenous Peoples (UNDRIP) 61/295:

Art.1. Indigenous peoples have the right to the full enjoyment, as a collective or as individuals, of all human rights and fundamental freedoms as recognized in the Charter of the United Nations, the Universal Declaration of Human Rights4 and international human rights law.
Art.3. Indigenous peoples have the right to self-determination. By virtue of that right they freely determine their political status and freely pursue their economic, social and cultural development.
Art.4. Indigenous peoples, in exercising their right to self-determination, have the right to autonomy or self-government in matters relating to their internal and local affairs, as well as ways and means for financing their autonomous functions.
Art.5. Indigenous peoples have the right to maintain and strengthen their distinct political, legal, economic, social and cultural institutions, while retaining their right to participate fully, if they so choose, in the political, economic, social and cultural life of the State.
Art.8.1. Indigenous peoples and individuals have the right not to be subjected to forced assimilation or destruction of their culture.
Art.10. Indigenous peoples shall not be forcibly removed from their lands or territories. No relocation shall take place without the free, prior and informed consent of the indigenous peoples concerned and after agreement on just and fair compensation and, where possible, with the option of return.
Art.11.1. Indigenous peoples have the right to practise and revitalize their cultural traditions and customs. This includes the right to maintain, protect and develop the past, present and future manifestations of their cultures, such as archaeological and historical sites, artefacts, designs, ceremonies, technologies and visual and performing arts and literature.
Art.11.2. States shall provide redress through effective mechanisms, which may include restitution, developed in conjunction with indigenous peoples, with respect to their cultural, intellectual, religious and spiritual property taken without their free, prior and informed consent or in violation of their laws, traditions and customs.
Art.14.1. Indigenous peoples have the right to establish and control their educational systems and institutions providing education in their own languages, in a manner appropriate to their cultural methods of teaching and learning.
Art.15.1. Indigenous peoples have the right to the dignity and diversity of their cultures, traditions, histories and aspirations which shall be appropriately reflected in education and public information.
Art.18. Indigenous peoples have the right to participate in decision-making in matters which would affect their rights, through representatives chosen by themselves in accordance with their own procedures, as well as to maintain and develop their own indigenous decision-making institutions.
Art.19. States shall consult and cooperate in good faith with the indigenous peoples concerned through their own representative institutions in order to obtain their free, prior and informed consent before adopting and implementing legislative or administrative measures that may affect them.
Art.20.1. Indigenous peoples have the right to maintain and develop their political, economic and social systems or institutions, to be secure in the enjoyment of their own means of subsistence and development, and to engage freely in all their traditional and other economic activities.
Art.21.1. Indigenous peoples have the right, without discrimination, to the improvement of their economic and social conditions, including, inter alia, in the areas of education, employment, vocational training and retraining, housing, sanitation, health and social security.
Art.21.2. States shall take effective measures and, where appropriate, special measures to ensure continuing improvement of their economic and social conditions. Particular attention shall be paid to the rights and special needs of indigenous elders, women, youth, children and persons with disabilities.
Art.22.1. Particular attention shall be paid to the rights and special needs of indigenous elders, women, youth, children and persons with disabilities in the implementation of this Declaration.
Art.26.1. Indigenous peoples have the right to the lands, territories and resources which they have traditionally owned, occupied or otherwise used or acquired.
Art.26.2. Indigenous peoples have the right to own, use, develop and control the lands, territories and resources that they possess by reason of traditional ownership or other traditional occupation or use, as well as those which they have otherwise acquired.
Art.26.3. States shall give legal recognition and protection to these lands, territories and resources. Such recognition shall be conducted with due respect to the customs, traditions and land tenure systems of the indigenous peoples concerned.
Art.27. States shall establish and implement, in conjunction with indigenous peoples a fair, independent, impartial, open and transparent process, giving due recognition to indigenous peoples' laws, traditions, customs and land tenure systems, to recognize and adjudicate the rights of indigenous peoples pertaining to their lands, territories and resources, including those which were traditionally owned or otherwise occupied or used.
Art.29.1. Indigenous peoples have the right to the conservation and protection of the environment and the productive capacity of their lands or territories and resources. States shall establish and implement assistance programmes for indigenous peoples for such conservation and protection, without discrimination.
Art.31.1. Indigenous peoples have the right to maintain, control, protect and develop their cultural heritage, traditional knowledge and traditional cultural expressions, as well as the manifestations of their sciences, technologies and cultures, including human and genetic resources, seeds, medicines, knowledge of the properties of fauna and flora, oral traditions, literatures, designs, sports and traditional games and visual and performing arts. They also have the right to maintain, control, protect and develop their intellectual property over such cultural heritage, traditional knowledge, and tradition- al cultural expressions.
Art.31.2. In conjunction with indigenous peoples, States shall take effective measures to recognize and protect the exercise of these rights.
Art.32. States shall consult and cooperate in good faith with the indigenous peoples concerned through their own representative institutions in order to obtain their free and informed consent prior to the approval of any project affecting their lands or territories and other resources, particularly in connection with the development, utilization or exploitation of mineral, water or other resources.
Art.38. States in consultation and cooperation with indigenous peoples, shall take the appropriate measures, including legislative measures, to achieve the ends of this Declaration.
Art.44. All the rights and freedoms recognized herein are equally guaranteed to male and female indigenous individuals.

Goals and Targets of the Sustainable Development Goals (SDGs):

Goal 2: End hunger, achieve food security and improved nutrition.
Target 2.1: End hunger and ensure access by all people, in particular the poor and people in vulnerable situations, including infants, to safe, nutritious and sufficient food all year round.
Goal 4: Ensure inclusive and equitable quality education and promote lifelong learning opportunities for all.
Target 4.5: Eliminate gender disparities in education and ensure equal access to all levels of education and vocational training for the vulnerable, including persons with disabilities, indigenous peoples and children in vulnerable situations
Target 4.6: By 2030, ensure that all youth and a substantial proportion of adults, both men and women, achieve literacy and numeracy
Target 4.7: Ensure that all learners acquire the knowledge and skills needed to promote sustainable development, including, among others, through education for sustainable development and sustainable lifestyles, human rights, gender equality, promotion of a culture of peace and non-violence, global citizenship and appreciation of cultural diversity and of culture's contribution to sustainable development.
Goal 5: Achieve gender equality and empower all women and girls.
Target 5.5: Ensure women's full and effective participation and equal opportunities for leadership at all levels of decision-making in political, economic and public life.
Target 5.A: Undertake reforms to give women equal rights to economic resources, as well as access to ownership and control over land and other forms of property, financial services, inheritance and natural resources, in accordance with national laws.
Target 5.C: Adopt and strengthen sound policies and enforceable legislation for the promotion of gender equality and the empowerment of all women and girls at all levels.
Target 9.5: Enhance scientific research, upgrade the technological capabilities of industrial sectors in all countries, in particular developing countries, including, by 2030, encouraging innovation and substantially increasing the number of research and development workers per 1 million people and public and private research and development spending
Goal 10: Reduce inequality within and among countries.
Target 10.3: Ensure equal opportunity and reduce inequalities of outcome, including by eliminating discriminatory laws, policies and practices and promoting appropriate legislation, policies and action in this regard.
Target 10.B: Encourage official development assistance and financial flows, including foreign direct investment, to States where the need is greatest, in particular least developed countries, African countries, small island developing States and landlocked developing countries, in accordance with their national plans and programmes.
Target 10.6: Ensure enhanced representation and voice for developing countries in decision-making in global international economic and financial institutions in order to deliver more effective, credible, accountable and legitimate institutions.
Goal 14: Conserve and sustainably use the oceans, seas and marine resources for sustainable development.
Target 14.2: Sustainably manage and protect marine and coastal ecosystems to avoid significant adverse impacts, including by strengthening their resilience, and take action for their restoration in order to achieve healthy and productive oceans.
Target 14.4: Effectively regulate harvesting and end overfishing, illegal, unreported and unregulated fishing and destructive fishing practices and implement science-based management plans, in order to restore fish stocks in the shortest time feasible, at least to levels that can produce maximum sustainable yield as determined by their biological characteristics.
Target 14.5: Conserve at least 10 per cent of coastal and marine areas, consistent with national and international law and based on the best available scientific information.
Target 14.7: Increase the economic benefits to Small Island developing States and least developed countries from the sustainable use of marine resources, including through sustainable management of fisheries, aquaculture and tourism.
Target 14.A: Increase scientific knowledge, develop research capacity and transfer marine technology, taking into account the Intergovernmental Oceanographic Commission Criteria and Guidelines on the Transfer of Marine Technology, in order to improve ocean health and to enhance the contribution of marine biodiversity to the development of developing countries, in particular small island developing States and least developed countries.
Target 17.2: Developed countries to implement fully their official development assistance commitments.
Target 17.3: Mobilize additional financial resources for developing countries from multiple sources.
Target 17.6: Enhance North-South, South-South and triangular regional and international cooperation on and access to science, technology and innovation and enhance knowledge sharing on mutually agreed terms, including through improved coordination among existing mechanisms, in particular at the United Nations level, and through a global technology facilitation mechanism.
Target 17.9: Enhance international support for implementing effective and targeted capacity-building in developing countries to support national plans to implement all the sustainable development goals, including through North-South, South-South and triangular cooperation.

CBDs' Aichi Biodiversity Targets 2010 (ABT):

Target 1: People are aware of the values of biodiversity and the steps they can take to conserve and use it sustainably.
Target 2: Biodiversity values have been integrated into national and local development and poverty reduction strategies and planning processes and are being incorporated into national accounting, as appropriate, and reporting systems.
Target 3: Incentives, including subsidies, harmful to biodiversity are eliminated, phased out or reformed in order to minimize or avoid negative impacts, and positive incentives for the conservation and sustainable use of biodiversity are developed and applied, consistent and in harmony with the Convention and other relevant international obligations, taking into account national socio economic conditions.
Target 4: Governments, business and stakeholders at all levels have taken steps to achieve or have implemented plans for sustainable production and consumption and have kept the impacts of use of natural resources well within safe ecological limits.
Target 5: The rate of loss of all natural habitats, including forests, is at least halved and where feasible brought close to zero, and degradation and fragmentation is significantly reduced.
Target 6: All fish and invertebrate stocks and aquatic plants are managed and harvested sustainably, legally and applying ecosystem based approaches, so that overfishing is avoided, recovery plans and measures are in place for all depleted species, fisheries have no significant adverse impacts on threatened species and vulnerable ecosystems and the impacts of fisheries on stocks, species and ecosystems are within safe ecological limits.
Target 10: The multiple anthropogenic pressures on coral reefs, and other vulnerable ecosystems impacted by climate change or ocean acidification are minimized, so as to maintain their integrity and functioning.

Target 11: At least 17 per cent of terrestrial and inland water, and 10 per cent of coastal and marine areas, especially areas of particular importance for biodiversity and ecosystem services, are conserved through effectively and equitably managed, ecologically representative and well connected systems of protected areas and other effective area-based conservation measures, and integrated into the wider landscapes and seascapes.
Target 14: Ecosystems that provide essential services, including services related to water, and contribute to health, livelihoods and well-being, are restored and safeguarded, taking into account the needs of women, indigenous and local communities, and the poor and vulnerable.
Target 15: Ecosystem resilience and the contribution of biodiversity to carbon stocks has been enhanced, through conservation and restoration, including restoration of at least 15 per cent of degraded ecosystems, thereby contributing to climate change mitigation and adaptation and to combating desertification.
Target 16: The Nagoya Protocol on Access to Genetic Resources and the Fair and Equitable Sharing of Benefits Arising from their Utilization is in force and operational, consistent with national legislation.
Target 17: Each Party has developed, adopted as a policy instrument, and has commenced implementing an effective, participatory and updated national biodiversity strategy and action plan.
Target 19: Knowledge, the science base and technologies relating to biodiversity, its values, functioning, status and trends, and the consequences of its loss, are improved, widely shared and transferred, and applied.
Target 20: At the latest, the mobilization of financial resources for effectively implementing the Strategic Plan for Biodiversity 2011-2020 from all sources, and in accordance with the consolidated and agreed process in the Strategy for Resource Mobilization, should increase substantially from the current levels. This target will be subject to changes contingent to resource needs assessments to be developed and reported by Parties.

Articles of the Indigenous and Tribal Peoples Convention C. 169 of the International Labour Organisation (ILO):

Art.2.1: Governments shall have the responsibility for developing, with the participation of the peoples concerned, co-ordinated and systematic action to protect the rights of these peoples and to guarantee respect for their integrity.
Art. 2.2(b): Promoting the full realisation of the social, economic and cultural rights of these peoples with respect for their social and cultural identity, their customs and traditions and their institutions;
Art. 2.2(c): Assisting the members of the peoples concerned to eliminate socio-economic gaps that may exist between indigenous and other members of the national community, in a manner compatible with their aspirations and ways of life.
Art.3.1: Indigenous and tribal peoples shall enjoy the full measure of human rights and fundamental freedoms without hindrance or discrimination. The provisions of the Convention shall be applied without discrimination to male and female members of these peoples.
Art.4.1: Special measures shall be adopted as appropriate for safeguarding the persons, institutions, property, labour, cultures and environment of the peoples concerned.
Art.5(a): The social, cultural, religious and spiritual values and practices of these peoples shall be recognised and protected, and due account shall be taken of the nature of the problems which face them both as groups and as individuals;
Art.5(b): The integrity of the values, practices and institutions of these peoples shall be respected;
Art.6(a): Consult the peoples concerned, through appropriate procedures and in particular through their representative institutions, whenever consideration is being given to legislative or administrative measures which may affect them directly;
Art.6(b): Establish means by which these peoples can freely participate, to at least the same extent as other sectors of the population, at all levels of decision-making in elective institutions and administrative and other bodies responsible for policies and programmes which concern them;
Art.6(c): Establish means for the full development of these peoples' own institutions and initiatives, and in appropriate cases provide the resources necessary for this purpose.
Art.7.1: The peoples concerned shall have the right to decide their own priorities for the process of development as it affects their lives, beliefs, institutions and spiritual well-being and the lands they occupy or otherwise use, and to exercise control, to the extent possible, over their own economic, social and cultural development. In addition, they shall participate in the formulation, implementation and evaluation of plans and programmes for national and regional development which may affect them directly.
Art.7.2: The improvement of the conditions of life and work and levels of health and education of the peoples concerned, with their participation and co-operation, shall be a matter of priority in plans for the overall economic development of areas they inhabit. Special projects for development of the areas in question shall also be so designed as to promote such improvement.
Art.7.3: Governments shall ensure that, whenever appropriate, studies are carried out, in cooperation with the peoples concerned, to assess the social, spiritual, cultural and environmental impact on them of planned development activities. The results of these studies shall be considered as fundamental criteria for the implementation of these activities.
Art.7.4: Governments shall take measures, in co-operation with the peoples concerned, to protect and preserve the environment of the territories they inhabit.
Art.8.1: In applying national laws and regulations to the peoples concerned, due regard shall be had to their customs or customary laws.
Art.8.2: These peoples shall have the right to retain their own customs and institutions, where these are not incompatible with fundamental rights defined by the national legal system and with internationally recognised human rights. Procedures shall be established, whenever necessary, to resolve conflicts which may arise in the application of this principle.
Art.13.1: In applying the provisions of this Part of the Convention Governments shall respect the special importance for the cultures and spiritual values of the peoples concerned of their relationship with the lands or territories, or both as applicable, which they occupy or otherwise use, and in particular the collective aspects of this relationship.

Art.13.2: The use of the term *lands* in Articles 15 and 16 shall include the concept of territories, which covers the total environment of the areas which the peoples concerned occupy or otherwise use.
Art.14.1: The rights of ownership and possession of the peoples concerned over the lands which they traditionally occupy shall be recognised. In addition, measures shall be taken in appropriate cases to safeguard the right of the peoples concerned to use lands not exclusively occupied by them, but to which they have traditionally had access for their subsistence and traditional activities. Particular attention shall be paid to the situation of nomadic peoples and shifting cultivators in this respect.
Art.14.2: Governments shall take steps as necessary to identify the lands which the peoples concerned traditionally occupy, and to guarantee effective protection of their rights of ownership and possession.
Art.14.3: Adequate procedures shall be established within the national legal system to resolve land claims by the peoples concerned.
Art.15.1: The rights to the natural resources pertaining their lands shall be specially safeguarded, including the right to participate in the use, management and conservation of these resources.
Art.15.2: In cases in which the State retains the ownership of sub-surface resources or rights to other resources pertaining to lands, governments shall establish or maintain procedures through which they consult tribal and indigenous, with a view to ascertaining whether and to what degree their interests would be prejudiced, before undertaking or permitting any programmes for the exploration or exploitation of such resources pertaining to their lands.
Art.16.1: Subject to the following paragraphs of this Article, the peoples concerned shall not be removed from the lands which they occupy.
Art.16.3: Whenever possible, these peoples shall have the right to return to their traditional lands, as soon as the grounds for relocation cease to exist.
Art.16.5: Persons thus relocated shall be fully compensated for any resulting loss or injury.
Art.22.3: Any special training programmes shall be based on the economic environment, social and cultural conditions and practical needs of the peoples concerned. Any studies made in this connection shall be carried out in co-operation with these peoples, who shall be consulted on the organisation and operation of such programmes. Where feasible, these peoples shall progressively assume responsibility for the organisation and operation of such special training programmes, if they so decide.
Art.23.1: Handicrafts, rural and community-based industries, and subsistence economy and traditional activities of the peoples concerned, such as hunting, fishing, trapping and gathering, shall be recognised as important factors in the maintenance of their cultures and in their economic self-reliance and development. Governments shall, with the participation of these people and whenever appropriate, ensure that these activities are strengthened and promoted.
Art.26.: Measures shall be taken to ensure that members of the peoples concerned have the opportunity to acquire education at all levels on at least an equal footing with the rest of the national community.
Art.27.1: Education programmes and services for the peoples concerned shall be developed and implemented in co-operation with them to address their special needs, and shall incorporate their histories, their knowledge and technologies, their value systems and their further social, economic and cultural aspirations.
Art.27.2: The competent authority shall ensure the training of members of these peoples and their involvement in the formulation and implementation of education programmes, with a view to the progressive transfer of responsibility for the conduct of these programmes to these peoples as appropriate.
Art.27.3: In addition, governments shall recognise the right of these peoples to establish their own educational institutions and facilities. Appropriate resources shall be provided for this purpose.
Article 32: Governments shall take appropriate measures, including by means of international agreements, to facilitate contacts and co-operation between indigenous and tribal peoples across borders, including activities in the economic, social, cultural, spiritual and environmental fields.

Articles of the International Covenant on Economic, Social and Cultural Rights:

Art.1.2: All peoples may, for their own ends, freely dispose of their natural wealth and resources without prejudice to any obligations arising out of international economic co-operation, based upon the principle of mutual benefit, and international law. In no case may a people be deprived of its own means of subsistence.
A.13.1: The States Parties to the present Covenant recognize the right of everyone to education.

Articles of the Declaration on the Rights of Persons Belonging to National or Ethnic, Religious and Linguistic Minorities:

Art.1.1: States shall protect the existence and the national or ethnic, cultural, religious and linguistic identity of minorities within their respective territories and shall encourage conditions for the promotion of that identity.
Art.1.2: States shall adopt appropriate legislative and other measures to achieve those ends.
Art.2.1: Persons belonging to national or ethnic, religious and linguistic minorities (hereinafter referred to as persons belonging to minorities) have the right to enjoy their own culture, to profess and practise their own religion, and to use their own language, in private and in public, freely and without interference or any form of discrimination.
Art.2.3: Persons belonging to minorities have the right to participate effectively in decisions on the national and, where appropriate, regional level concerning the minority to which they belong or the regions in which they live according with national legislation.
Art.5.1: National policies and programmes shall be planned and implemented with due regard for the legitimate interests of persons belonging to minorities.
Art.5.2: Programmes of cooperation and assistance among States should be planned and implemented with due regard for the legitimate interests of persons belonging to minorities.
